# Comprehensive biomarker profiling of hypertension in 36 985 Finnish individuals

**DOI:** 10.1097/HJH.0000000000003051

**Published:** 2021-11-15

**Authors:** Joonatan Palmu, Emmi Tikkanen, Aki S. Havulinna, Erkki Vartiainen, Annamari Lundqvist, Matti O. Ruuskanen, Markus Perola, Mika Ala-Korpela, Pekka Jousilahti, Peter Würtz, Veikko Salomaa, Leo Lahti, Teemu Niiranen

**Affiliations:** a Department of Medicine, Turku University Hospital and University of Turku, Turku; b Department of Public Health and Welfare, Finnish Institute for Health and Welfare; c Nightingale Health Plc; d Institute for Molecular Medicine Finland (FIMM), HiLIFE, Helsinki; e Department of Computing, University of Turku, Turku, Finland; f Estonian Genome Center, University of Tartu, Tartu, Estonia; g Institute for Molecular Medicine, University of Helsinki, Helsinki; h Computational Medicine, Faculty of Medicine, University of Oulu and Biocenter Oulu; i Center for Life Course Health Research, University of Oulu, Oulu; j NMR Metabolomics Laboratory, School of Pharmacy, University of Eastern Finland, Kuopio, Finland

**Keywords:** amino acids, blood pressure, hypertension, inflammation, lipids, nuclear magnetic resonance spectroscopy

## Abstract

Previous studies on the association between metabolic biomarkers and hypertension have been limited by small sample sizes, low number of studied biomarkers, and cross-sectional study design. In the largest study to date, we assess the cross-sectional and longitudinal associations between high-abundance serum biomarkers and blood pressure (BP).

**Methods:**

We studied cross-sectional (*N* = 36 985; age 50.5 ± 14.2; 53.1% women) and longitudinal (*N* = 4197; age 49.4 ± 11.8, 55.3% women) population samples of Finnish individuals. We included 53 serum biomarkers and other detailed lipoprotein subclass measures in our analyses. We studied the associations between serum biomarkers and BP using both conventional statistical methods and a machine learning algorithm (gradient boosting) while adjusting for clinical risk factors.

**Results:**

Fifty-one of 53 serum biomarkers were cross-sectionally related to BP (adjusted *P* < 0.05 for all). Conventional linear regression modeling demonstrated that LDL cholesterol, remnant cholesterol, apolipoprotein B, and acetate were positively, and HDL particle size was negatively, associated with SBP change over time (adjusted *P* < 0.05 for all). Adding serum biomarkers (cross-sectional root-mean-square error: 16.27 mmHg; longitudinal: 17.61 mmHg) in the model with clinical measures (cross-sectional: 16.70 mmHg; longitudinal 18.52 mmHg) improved the machine learning model fit. Glucose, albumin, triglycerides in LDL, glycerol, VLDL particle size, and acetoacetate had the highest importance scores in models related to current or future BP.

**Conclusion:**

Our results suggest that serum lipids, and particularly LDL-derived and VLDL-derived cholesterol measures, and glucose metabolism abnormalities are associated with hypertension onset. Use of serum metabolite determination could improve identification of individuals at high risk of developing hypertension.

## INTRODUCTION

Although many hypertension risk factors have been identified, the exact mechanisms behind the age-related increase in blood pressure (BP) remain elusive. Present-day metabolomics offers a promising method to identify and study metabolic biomarkers associated with current hypertension and future hypertension onset. However, only a few prior studies have reported associations between a large number of serum metabolic measures and hypertension in large population cohorts with a longitudinal design [1]. Therefore, additional research on the cross-sectional and longitudinal associations between metabolic biomarkers and hypertension in large population samples are warranted. This information could potentially be used to elucidate the metabolic underpinnings of elevated BP [2].

Recent development in high-throughput nuclear magnetic resonance (NMR) spectroscopy-based metabolic profiling offers a method for studying advanced lipid measures, fatty acids, amino acids, and inflammatory proteins in large population samples [2,3]. In the present study, we aim to advance the current knowledge on the associations between circulating metabolic biomarkers and BP in well phenotyped, representative cross-sectional and longitudinal population samples of 36 985 and 4197 participants, respectively.

## METHODS

### Cross-sectional study sample

Our study sample consists of six Finnish population samples, FINRISK 1997, FINRISK 2002, FINRISK 2007, FINRISK 2012, Health 2000, and FinHealth 2017 (Fig. S1). All studies are coordinated by the Finnish Institute for Health and Welfare. The studies were approved by the Coordinating Ethics Committee of the Helsinki University Hospital District and all participants gave written informed consent.

The FINRISK studies have been performed every 5 years since 1972 to monitor the development of cardiovascular risk factors in Finnish population aged 25–74 years [4]. The FINRISK 1997–2012 study samples consist of participants randomly drawn from the national population register from up to six geographical areas in Finland. The proportion of individuals who participated in the health examinations varied between 56 and 72% [4]. Health 2000 is a multidisciplinary epidemiological survey of individuals aged at least 30 years living in mainland Finland [5]. The participants, living in 80 municipalities around Finland, were randomly drawn from the population register in the year 2000 to participate in a health examination. The participation rate for health examination was 83%. In FinHealth 2017 study [6], participants aged at least 18 years were drawn from the national population register for municipalities in mainland Finland to participate in a health examination. The participation rate of the health examination was 58%.

Data were available for a total of 38404 FINRISK, Health 2000, and FinHealth 2017 participants. We excluded 1419 participants because of missing covariates for a final study sample of 36 985 individuals who were included in the analysis (Fig. S1).

### Longitudinal study sample

FINRISK 2007 participants who lived in Helsinki, Vantaa, Turku and Loimaa area were invited to a follow-up examination in 2014 (participation rate for health examination in 2014 54%) [7,8]. All living participants of Health 2000 were invited to participate in a follow-up examination in 2011 (participation rate for health examination 59%) [9]. Data were available for 968 FINRISK 2007 and 3229 Health 2000 participants with repeated BP measurements for a final longitudinal study sample of 4197 participants (Fig. S1).

### Study flow

In the FINRISK and FinHealth 2017 studies, after filling in a questionnaire on sociodemographic information, lifestyles, medications, and medical history at home, the participants attended a physical examination at a local study site. The participants underwent measurements for height and weight and blood samples were drawn mainly after a minimum of 4 h of fasting [4]. A study nurse measured sitting BP three times from the right arm using a mercury sphygmomanometer with an appropriately sized cuff.

In the Health 2000–2011 study, participants were interviewed by centrally trained interviewers on sociodemographic information, lifestyle, medications, and medical history 1–6 weeks before attending physical examination at local study sites. The participants underwent measurements for height and weight. Overnight fasting blood samples were drawn. A study nurse measured sitting BP two times from the right arm using a mercury sphygmomanometer and a 15 × 43 cm sized cuff; a larger cuff was used when needed.

### Serum samples and storage

In all studies, samples were delivered in dry ice to the Finnish National Institute for Health and Welfare and stored at −70 °C [5,10]. Metabolic analyses were performed using 1H-NMR spectroscopy on highly automated platform (Nightingale Health Plc, Helsinki, Finland; biomarker quantification version 2016) [3]. In short, 350 μm serum aliquots were quantified in molar concentration units independently from other samples in the same well plate or same cohort [11]. Measured NMR concentrations have been reported to be consistent with the available clinical chemistry assays (cholesterol measures, apolipoproteins, total triglycerides, glucose, creatinine, and albumin) performed soon after sample collection [10]. The utilized NMR metabolomics technology has received regulatory approval (CE) and 37 biomarkers in the panel have been certified to diagnostic use [11]. We included in our core analyses 53 circulating biomarkers and also studied 97 lipoprotein measures related to 14 lipoprotein subclasses.

### Outcome variables and covariates

The mean of the last two BP measurements was used to determine SBP and DBP. Hypertension was defined as SBP at least 140 mmHg, DBP at least 90 mmHg, or self-reported use of antihypertensive medication. BMI was defined as weight divided by the square of the body height. Use of lipid medication, diabetes, smoking, and leisure-time physical activity were self-reported. Leisure-time activity was divided into four categories: sedentary, light activity for over 4 h per week, fitness training or other strenuous exercise for over 3 h per week, and competitive sports. Smoking was defined as daily use of tobacco products.

### Statistical methods

All metabolic biomarkers (including percentages) were centered to zero and standardized to unit variance (Table S1 and Table S2) to simplify pair-wise comparisons and graphical representation of the results. Unless otherwise noted, we adjusted all analyses for age, sex, BMI, smoking, diabetes mellitus, leisure-time physical activity, antihypertensive medication (unless the dependent variable was hypertension), lipid medication, and cohort. We studied the associations for baseline BP and SBP change in follow-up with baseline metabolic measures using linear and logistic regression models. We adjusted analyses for multiple testing using Benjamini–Hochberg correction [12]. The SBP, and not DBP was studied in longitudinal analysis because of its linear relation with age. We also performed the previous analyses stratified by median age (50.5 years) and sex. To assess the validity of sample pooling, we also performed an inverse variance-weighted fixed-effect meta-analysis for per cohort results in cross-sectional (six cohorts) and longitudinal (two cohorts) study samples.

In addition to conventional (univariable) statistical approaches, we assessed the multivariable associations of the 53 circulating biomarkers with baseline and follow-up SBP using a XGBoost gradient boosting machine learning algorithm. First, we used three sets of model covariates to assess model fit: only clinical characteristics, only metabolic measures, and the combination of clinical characteristics and metabolic measures [13]. In the longitudinal models, baseline SBP was included in among the covariates. We trained our cross-sectional model using a leave-one-group-out cross-validation in FINRISK 1997–2002 and Health 2011 and used FinHealth 2017 for testing. We used root-mean-square error to measure training fit in cross-validation. In the longitudinal analyses, we used the Health 2000–2011 cohort for training with five-fold cross-validation and the FINRISK 2007–2014 cohort for testing. We performed Bayesian optimization with the R package ‘mlrMBO’ to tune the hyperparameters using 42 (six times the number of hyperparameters) preliminary rounds followed by 100 optimization rounds [14]. We constructed partial dependency plots with the R package ‘pdp’ to study and visualize the (marginal) associations between model covariates and SBP [15]. We estimated the fit of final gradient boosting models using the root-mean-square error.

We used R version 3.6.1 for all statistical analyses and the source code for the analyses is openly available at doi:10.5281/zenodo.3625488 [16,17].

## RESULTS

The characteristics of the cross-sectional (*N* = 36 985, mean age 50.5 ± 14.2 years, 53.1% women) and longitudinal (*N* = 4197, mean age at baseline 49.4 ± 11.8 years, 55.3% women) study samples are reported in Table 1 and Table S3, respectively. The sample selection flow is presented in Fi. S1.

**TABLE 1:** Characteristics of the cross-sectional study sample

Characteristics	Total	FINRISK 1997	Health 2000	FINRISK 2002	FINRISK 2007	FINRISK 2012	FINRISK 2017
*N*	36 985	7106	6039	7565	5825	5420	5030
Age (years) (SD)	50.5 (14.2)	48.2 (13.1)	52.4 (14.7)	47.9 (13.1)	50.9 (13.9)	51.2 (14.0)	54.1 (16.2)
Female [*N* (%)]	19652 (53.1)	3585 (50.5)	3293 (54.5)	4180 (55.3)	3071 (52.7)	2821 (52.0)	2702 (53.7)
BMI (kg/m^2^) (SD)	27.0 (4.7)	26.6 (4.5)	26.9 (4.6)	26.8 (4.6)	27.1 (4.8)	27.1 (4.9)	27.5 (5.0)
SBP (mmHg) (SD)	134.7 (19.9)	135.6 (19.8)	134.6 (21.0)	134.5 (19.9)	135.8 (20.4)	133.9 (18.8)	133.8 (19.3)
DBP (mmHg) (SD)	80.4 (11.3)	82.3 (11.3)	81.8 (11.2)	78.7 (11.3)	78.9 (11.4)	81.2 (11.0)	79.3 (11.1)
Hypertension [*N* (%) ]	17424 (47.1)	3328 (46.8)	2838 (47.0)	3242 (42.9)	2855 (49.0)	2668 (49.2)	2493 (49.6)
Current smoker [*N* (%)]	8003 (21.6)	1702 (24.0)	1312 (21.7)	1951 (25.8)	1196 (20.5)	1038 (19.2)	804 (16.0)
Diabetes mellitus [*N* (%)]	1748 (4.7)	209 (2.9)	325 (5.4)	211 (2.8)	238 (4.1)	341 (6.3)	424 (8.4)
Exercise [*N* (%)]							
Light	8502 (23.0)	1604 (22.6)	1652 (27.4)	1697 (22.4)	1188 (20.4)	1145 (21.1)	1216 (24.2)
Moderate	19550 (52.9)	4044 (56.9)	3329 (55.1)	4088 (54.0)	3115 (53.5)	2640 (48.7)	2334 (46.4)
Heavy	8392 (22.7)	1376 (19.4)	980 (16.2)	1694 (22.4)	1426 (24.5)	1529 (28.2)	1387 (27.6)
Competitive	541 (1.5)	82 (1.2)	78 (1.3)	86 (1.1)	96 (1.6)	106 (2.0)	93 (1.8)
Antihypertensive medication [*N* (%)]	6799 (18.4)	927 (13.0)	1068 (17.7)	1073 (14.2)	1200 (20.6)	1215 (22.4)	1316 (26.2)
Lipid medication [*N* (%)]	3632 (9.8)	237 (3.3)	374 (6.2)	534 (7.1)	827 (14.2)	834 (15.4)	826 (16.4)

Continuous variables are presented as mean (standard deviation) and categorical values as count (percentage). BP, blood pressure.

**FIGURE 1:**
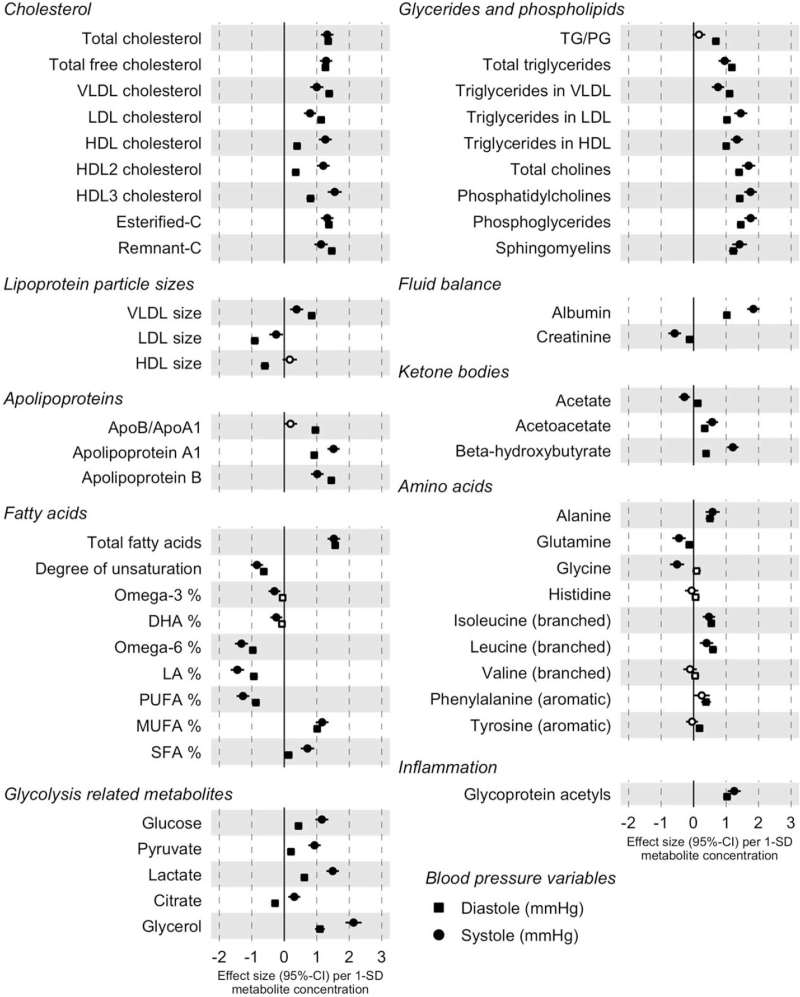
Cross-sectional associations between the metabolites and blood pressure (*N* = 36 985). Filled circle signifies FDR-corrected *P* less than 0.05. Associations are adjusted for age, sex, BMI, current smoking, diabetes, antihypertensive medication, exercise, lipid medication, and cohort. Apo, apolipoprotein; C, cholesterol; DHA, docosahexaenoic acid; HDL, high-density lipoprotein; LA, linoleic acid; LDL, low-density lipoprotein; MUFA, monounsaturated fatty acids; PG, phosphoglycerides; PUFA, polyunsaturated fatty acids; SFA, saturated fatty acids; TG, triglycerides; VLDL, very-low density lipoprotein.

In the conventional cross-sectional analyses performed using linear and logistic regression models, only two amino acids (histidine and valine) of all 53 circulating biomarkers included in our analysis were not associated with BP (Fig. 1, Table S4). Six metabolic measures, docosahexaenoic acid, citrate, creatinine, histidine, valine, and tyrosine, were not associated with hypertension (Fig. S2, Table S4). Stratified analysis by sex (Fig. S3, Table S5) and median age (Fig. S4, Table S6) were highly consistent; men compared with women and younger participants compared to older participants had in some cases slightly larger effect sizes. However, acetate was negatively associated with hypertension in women only and acetoacetate positively associated with hypertension in men only. In older than median age participants, total cholesterol, low-density lipoprotein (LDL) cholesterol, esterified cholesterol, and high-density lipoprotein (HDL) cholesterol were not associated with hypertension. Large and extremely large HDL fractions were negatively and medium and small HDL fractions positively associated with hypertension (Fig. S5)

**FIGURE 2:**
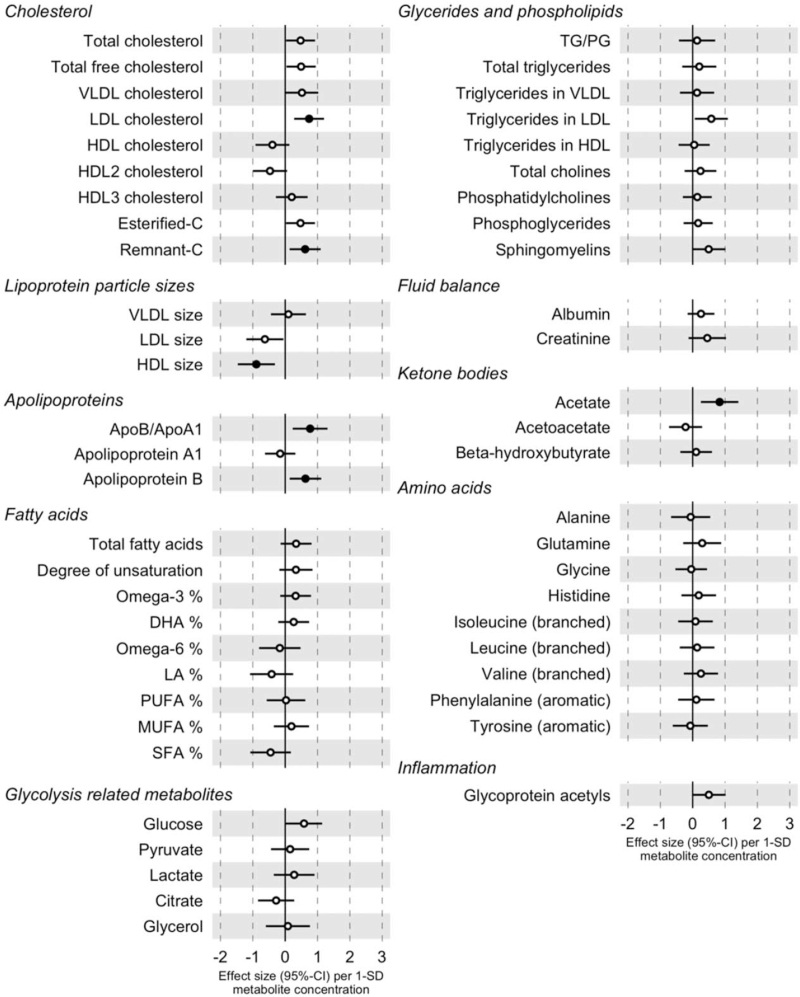
Longitudinal associations between baseline metabolites levels and SBP change (*N* = 4197). Filled circle signifies FDR-corrected *P* less than 0.05. Associations are adjusted for baseline SBP, age, sex, BMI, current smoking, diabetes, antihypertensive medication, exercise, lipid medication, and cohort. Apo, apolipoprotein; C, cholesterol; DHA, docosahexaenoic acid; HDL, high-density lipoprotein; LA, linoleic acid; LDL, low-density lipoprotein; MUFA, monounsaturated fatty acids; PG, phosphoglycerides; PUFA, polyunsaturated fatty acids; SFA, saturated fatty acids; TG, triglycerides; VLDL, very low-density lipoprotein.

In the longitudinal analyses with conventional statistical approaches (*N* = 4197), we examined the associations between baseline metabolites and change in SBP between baseline and follow-up of 7–11 years (Fig. 2, Table S7). We observed that LDL cholesterol [*β* = 0.74 mmHg per 1SD normalized concentration; 95% confidence interval (CI) 0.28–1.20 mmHg; *P* = 0.01], remnant cholesterol [*β* = 0.62 mmHg; 95% CI 0.14–1.10 mmHg; *P* = 0.03], apolipoprotein B [*β* = 0.63 mmHg; 95% CI 0.14–1.11 mmHg; *P* = 0.03], and acetate [*β* = 0.83 mmHg; 95% CI 0.25–1.41 mmHg; *P* = 0.02] were associated with a BP increase and average HDL particle size [*β*=-0.89; 95% CI −1.46 to −0.32 mmHg; *P* = 0.01) with a BP decrease during follow-up. Large and extremely large HDL fractions were negatively and other lipoprotein fractions (VLDL, IDL, and LDL) mostly positively associated with SBP change in follow-up (Fig. S6).

The observed cross-sectional associations were consistent across study cohorts (Fig. S7). The results from inverse variance-weighted fixed-effect meta-analysis were also consistent with the pooled cross-sectional (Fig. S8) and longitudinal (Fig. S9) analyses.

We then examined the overall relation of the metabolic biomarkers with current and future SBP using multivariable gradient boosting machine learning algorithm. First, we compared the predictive accuracy between models that included only clinical characteristics, only metabolic measures, and both the clinical variables and metabolic measures. The hyperparameters used in models are presented in Table S8. Adding metabolic measures (cross-sectional root-mean-square error: 16.27 mmHg; longitudinal: 17.61 mmHg) in the model with clinical measures (cross-sectional: 16.70 mmHg; longitudinal 18.52 mmHg) improved the machine learning model fit (Table 2).

**TABLE 2:** Measurement of gradient boosting mode fit in cross-sectional and longitudinal samples

		Root-mean-square error		
Sample	Step	Full model	Clinical characteristics	Metabolic measures
Cross-sectional	Training	15.50 mmHg	16.95 mmHg	16.36 mmHg
Cross-sectional	Test	16.27 mmHg	16.70 mmHg	18.03 mmHg
Longitudinal	Training	13.22 mmHg	14.84 mmHg	16.46 mmHg
Longitudinal	Test	17.61 mmHg	18.52 mmHg	19.78 mmHg

Full model included both clinical characteristics and 53 circulating metabolic biomarkers. In models adjusted with clinical characteristics, we used following covariates age, sex, BMI, current smoking, diabetes, antihypertensive medication, exercise, and lipid medication, and baseline SBP (only in longitudinal model).

**FIGURE 3:**
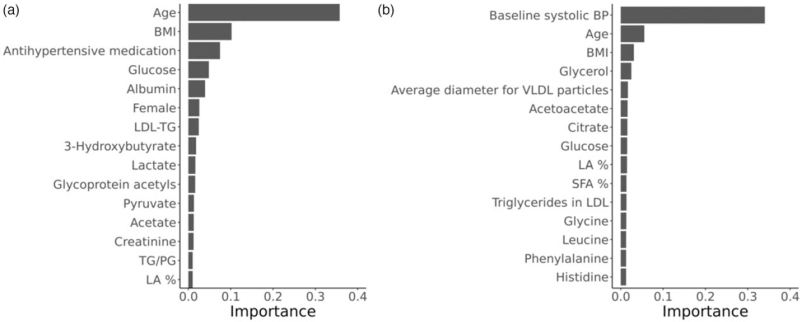
The top 15 clinical and metabolic measures of current (a) and future (b) blood pressure assessed using a multivariable machine learning approach (gradient boosting). The importance (gain) of a feature represents the total contribution each feature has in the trees of the gradient boosting model. In addition to the 53 metabolic measures, the model was adjusted for age, sex, BMI, current smoking, diabetes, antihypertensive medication, exercise, and lipid medication, and baseline SBP (only in longitudinal model). The sum of the importance of all the features equals one. LA, linoleic acid; LDL, low-density lipoprotein; PG, phosphoglycerides; TG, triglycerides; SFA, saturated fatty acids.

**FIGURE 4:**
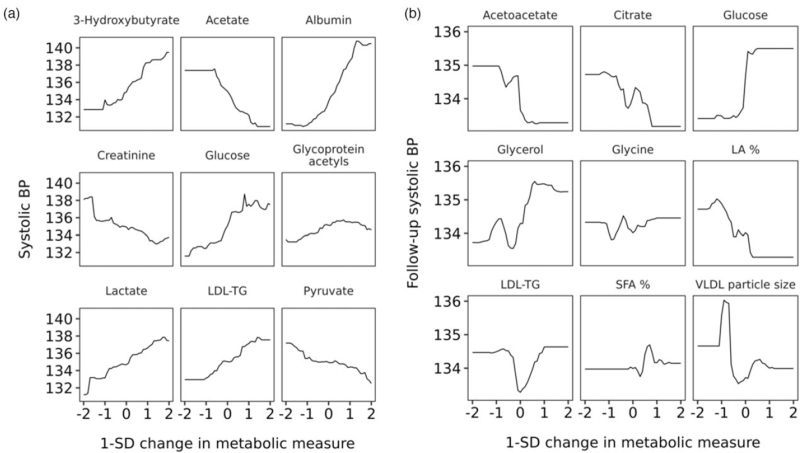
The multivariable-adjusted relation between the top nine metabolites and current (a) and future (b) blood pressure. The top nine biomarkers in the gradient boosting models are represented for cross-sectional (a) and longitudinal (b) samples. Partial dependency plots approximate the relationship between single metabolic measure and outcome variable when the effect of all other model covariates is averaged out. LA, linoleic acid; LDL, low-density lipoprotein; TG, triglycerides; SFA, saturated fatty acids.

Then, we assessed the independent associations of the metabolic measures with current and future SBP using the same machine learning approach. Expectedly, the clinical characteristics, such as age, BMI, antihypertensive medication (cross-sectional model), and baseline SBP (longitudinal model) contributed the most to the predictive ability of the models (Fig. 3). Of the metabolic measures, glucose, albumin, and triglycerides in LDL were the most important metabolic measures in the cross-sectional model. In the longitudinal model, the three most important metabolic predictors of future SBP were glycerol, average VLDL size, and acetoacetate, which were then followed by other metabolic measures with similar levels of importance. The multivariable-adjusted association between the most important metabolic measures and current or future BP is shown in Fig. 4.

## DISCUSSION

We investigated the cross-sectional and longitudinal associations between serum high-abundance metabolic measures and BP in representative population samples of up to 36 985 and 4197 individuals. In addition to conventional statistical methods where a single metabolic measure was assessed in each model, we used a machine learning approach to identify a metabolic signature related to blood pressure and longitudinal blood pressure change. Our results demonstrate that metabolic measures provide incremental predictive value over conventional clinical variables for current and future SBP. In cross-sectional models, glucose, albumin, and triglycerides in LDL were the strongest metabolic correlates of SBP. In longitudinal models, glycerol, average VLDL size, and acetoacetate were the strongest predictors of future SBP. We did not observe any major sex-specific or age-specific differences between the studied 53 metabolic measures and BP.

In our study, triglycerides in LDL were strongly associated with current BP whereas average VLDL was a robust predictor of future BP in the machine learning models. The longitudinal associations between circulating lipoprotein fractions and hypertension have been studied by certain population studies. In the Brisighella Heart Study (*N* = 1864), baseline LDL cholesterol level was positively related to rate of new-onset of hypertension [18]. In the Women's Health Study (*N* = 17 527), average VLDL particle size, as in our study, was positively associated with the development of hypertension. In that study, apolipoprotein B and total triglycerides were also predictive of future hypertension whereas average LDL particle size was negatively associated with the development of hypertension [19]. The association between HDL fractions and hypertension, however, appears to be more convoluted. In the Women's Health Study, medium-HDL and small-HDL particle concentrations were positively associated with future hypertension. In contrast, average HDL particle size, large-HDL particle concentration, and HDL cholesterol were negatively associated with the development of hypertension [19]. In a cross-sectional study of 14 215 normotensive men, baseline HDL cholesterol categorized in five groups demonstrated a U-shaped association with the development of hypertension [20]. HDL particles isolated from patients with cardiovascular disease have been reported to lack the ability to induce endothelial nitric oxide production and stimulate endothelial repair [21,22]. All in all, results from our and previous studies suggest that serum lipids, and particularly LDL-derived and VLDL-derived cholesterol levels are associated with the development of hypertension.

In the multivariable machine learning model, glycolysis-related metabolic markers glucose and glycerol were associated with BP. In healthy individuals, postprandial insulin secretion related to increasing glucose levels promotes four changes in circulating metabolites reflecting switch from catabolism to anabolism: increased glycolysis (lactate increases) and decreased lipolysis (glycerol decreases), and ketogenesis (beta-hydroxybutyrate and acetoacetate decrease) [23]. Two prior studies have reported on the associations of glycolysis-related metabolic measures, including ketone bodies, with hypertension in humans. In the Bogalusa Heart Study (*N* = 1249), fasting glucose was positively associated with SBP [24]. In an American prospective study (*N* = 5554), lactate, an indicator of oxidative capacity, was positively associated with development of hypertension in women but not in men [25]. As insulin resistance and diabetes are both related to arterial stiffening, these findings are somewhat expected and highlight the importance of glucose metabolism abnormalities in the development of hypertension [26].

In the cross-sectional univariate and multivariable analyses, we observed a positive association between albumin and blood pressure. Albumin is a key contributor to the vascular colloid-osmotic pressure and important transporter of hormones, drugs, amino acids, and free fatty acids [27]. In the Oslo Health Study (*N* = 5171) and in the Neuroprotective Model for Healthy Longevity among the Malaysian Elderly study (*N* = 2322), positive cross-sectional associations were observed between albumin and BP [27,28]. However, a retrospective study of normotensive Japanese (*N* = 2240) reported a negative association between blood albumin and risk of hypertension [29]. The authors of this study concluded that the positive cross-sectional associations could be explained by increased vascular volume whereas the negative longitudinal associations could be a result of the anti-inflammatory and antioxidant properties of albumin [29]. As albumin was not a significant correlate of future BP, our cross-sectional findings mainly suggest that the association between albumin and BP may not be causal.

Although nearly all measured fatty acids and amino acids were strongly related to BP in the cross-sectional analyses, none of them demonstrated any strong associations in the longitudinal analyses. Despite several prior studies reporting strong cross-sectional associations of fatty acids and amino acids with BP [30–35], the nature and strength of these associations warrants further study.

### Strengths and limitations

Our study has several strengths, such as a large cross-sectional population sample, access to repeated measurements, and consistent biomarker quantification by the same NMR spectrometry method in all included cohorts. However, our results must be interpreted in the context of potential limitations. First, although feasible to perform in large scale, NMR provided only a limited number of metabolic measures, of which the majority were lipid measures. Second, our longitudinal sample size, although relatively large, may have been insufficient to capture some associations. Third, the dates of baseline examinations ranged over 15 years in our study cohorts resulting differences in freezing times. Fourth, many of the statistically significant associations that were observed had relatively small effect sizes.

In conclusion, we assessed the metabolic profile of hypertension in a representative population sample of up to 36 985 individuals with repeated measurements available for 4197 participants. Our study is the largest study to date to investigate the relation between circulating metabolic measures and hypertension. We identified a metabolic serum signature associated with blood pressure and longitudinal blood pressure change using conventional statistics and machine learning approaches. Our results suggest that serum lipids, and particularly LDL- and VLDL-derived cholesterol levels, and glucose metabolism abnormalities are associated with hypertension onset. Use of serum NMR metabolite determination could improve the identification of individuals at high risk of developing hypertension.

## ACKNOWLEDGEMENTS

We thank the participants and staff of the FINRISK 1997–2012, Health 2000–2011, and FinHealth 2017 studies.

Availability of data and materials: the data that support the findings of this study are available from Finnish Institute for Health and Welfare Biobank (https://thl.fi/en/web/thl-biobank). The source code for the analyses is openly available at doi:10.5281/zenodo.3625488.

Previous presentations: the summary of the study was presented in poster session of the conference of American College of Cardiology (ACC.21) 15–17 May 2021.

### Conflicts of interest

V.S. has received honoraria from Novo Nordisk and Sanofi for consultations and travel support from Novo Nordisk. He also has ongoing research collaboration with Bayer Ltd. (all unrelated to the present study). E.T. and P.W. are shareholders and/or employees of Nightingale Health, Plc, a company offering nuclear magnetic resonance-based metabolic profiling. This work was supported by the Emil Aaltonen Foundation (T.N.), the Paavo Nurmi Foundation (J.P., T.N.), the Finnish Medical Foundation (T.N.), the Finnish Foundation for Cardiovascular Research (V.S.), the Academy of Finland (grant no. 321351 to T.N.; 295741, 307127 to L.L.; 321356 to A.H.; 338818 to M.R.), and the Sigrid Juselius Foundation (M.A.K.). Although Nightingale Health Plc. funded and performed the serum biomarker measurements, the funders play no further role in the design of the study, the collection, analysis, and interpretation of the data; and the decision to approve publication of the finished manuscript. All authors had full access to all of the data (including statistical reports and tables) in the study and can take responsibility for the integrity of the data and the accuracy of the data analysis.

## Supplementary Material

Supplemental Digital Content
