# Supplementary material for: Comprehensive biomarker profiling of hypertension in 36 985 Finnish individuals
Source: J Hypertens. 2021 Nov 15;40(3):579–87. doi: 10.1097/HJH.0000000000003051 (PMC8815836; doi:10.1097/HJH.0000000000003051)
Supplement: Supplemental Digital Content [file jhype-40-579-s001.docx]

**SUPPLEMENTAL MATERIAL**

**Metabolic Profiling of Hypertension in 36985 Finnish Individuals**

Authors:

Joonatan PALMU MD^a,b^, Emmi TIKKANEN PhD^c^, Aki S. HAVULINNA DSc (Tech)^b,d^, Erkki VARTIAINEN MD, PhD^b^, Annamari LUNDQVIST PhD^b^, Matti O. RUUSKANEN PhD^e^, Markus PEROLA MD, PhD^b,f,g^, Mika ALA-KORPELA PhD^h,i,j^, Pekka JOUSILAHTI MD, PhD^b^, Peter WÜRTZ PhD^c^, Veikko SALOMAA MD, PhD^b^, Leo LAHTI DSc (Tech)^e^, Teemu NIIRANEN MD, PhD^a,b^

^a^Department of Medicine, Turku University Hospital and University of Turku, Turku, Finland

^b^Department of Public Health Solutions, Finnish Institute for Health and Welfare, Helsinki, Finland

^c^Nightingale Health Plc., Helsinki, Finland

^d^Institute for Molecular Medicine Finland (FIMM) and Helsinki Institute of Life Science (HiLIFE) Helsinki Finland

^e^Department of Computing, University of Turku, Turku, Finland

^f^Estonian Genome Center, University of Tartu, Tartu 51010, Estonia.

^g^Institute for Molecular Medicine, University of Helsinki, FI-00014 Helsinki, Finland.

^h^Computational Medicine, Faculty of Medicine, University of Oulu and Biocenter Oulu, Oulu, Finland

**Supplemental Tables and Figures**

**Table S1.** Characteristics of the studied metabolic measures.

| Abbreviation | Description | Metabolite group | N | Mean | SD | Unit |
| --- | --- | --- | --- | --- | --- | --- |
| Acetate | Acetate | Ketone bodies | 36981 | 0.048 | 0.036 | mmol/l |
| Acetoacetate | Acetoacetate | Ketone bodies | 36895 | 0.060 | 0.044 | mmol/l |
| Alanine | Alanine | Amino acids | 36977 | 0.370 | 0.067 | mmol/l |
| Albumin | Albumin | Fluid balance | 36984 | 0.092 | 0.009 | signal area |
| ApoB/ApoA1 | Ratio of apolipoprotein B to apolipoprotein A1 | Apolipoproteins | 36981 | 0.586 | 0.169 | ratio |
| Apolipoprotein A1 | Apolipoprotein A1 | Apolipoproteins | 36984 | 1.550 | 0.224 | g/l |
| Apolipoprotein B | Apolipoprotein B | Apolipoproteins | 36981 | 0.894 | 0.240 | g/l |
| Beta-hydroxybutyrate | Beta-hydroxybutyrate | Ketone bodies | 36889 | 0.200 | 0.138 | mmol/l |
| Citrate | Citrate | Glycolysis related metabolites | 36971 | 0.116 | 0.020 | mmol/l |
| Creatinine | Creatinine | Fluid balance | 36703 | 0.060 | 0.013 | mmol/l |
| Degree of unsaturation | Degree of unsaturation | Fatty acids | 36855 | 1.221 | 0.073 | degree |
| DHA % | Ratio of docosahexaenoic acid to total fatty acids | Fatty acids | 36852 | 1.449 | 0.471 | % |
| Esterified-C | Total esterified cholesterol | Cholesterol | 36905 | 3.255 | 0.792 | mmol/l |
| Glucose | Glucose | Glycolysis related metabolites | 36962 | 4.130 | 0.923 | mmol/l |
| Glutamine | Glutamine | Amino acids | 35990 | 0.500 | 0.080 | mmol/l |
| Glycerol | Glycerol | Glycolysis related metabolites | 28978 | 0.101 | 0.054 | mmol/l |
| Glycine | Glycine | Amino acids | 36933 | 0.248 | 0.066 | mmol/l |
| Glycoprotein acetyls | Glycoprotein acetyls | Inflammation | 36981 | 1.287 | 0.256 | mmol/l |
| HDL cholesterol | HDL cholesterol | Cholesterol | 36984 | 1.493 | 0.391 | mmol/l |
| HDL size | Average diameter for HDL particles | Lipoprotein particle sizes | 36984 | 10.016 | 0.302 | nm |
| HDL2 cholesterol | HDL2 cholesterol | Cholesterol | 36885 | 0.988 | 0.358 | mmol/l |
| HDL3 cholesterol | HDL3 cholesterol | Cholesterol | 36984 | 0.507 | 0.047 | mmol/l |
| Histidine | Histidine | Amino acids | 36970 | 0.065 | 0.011 | mmol/l |
| Isoleucine | Isoleucine | Amino acids | 36960 | 0.055 | 0.020 | mmol/l |
| LA % | Ratio of linoleic acid to total fatty acids | Fatty acids | 36855 | 26.511 | 3.495 | % |
| Lactate | Lactate | Glycolysis related metabolites | 36982 | 1.155 | 0.301 | mmol/l |
| LDL cholesterol | LDL cholesterol | Cholesterol | 36978 | 1.695 | 0.573 | mmol/l |
| LDL size | Average diameter for LDL particles | Lipoprotein particle sizes | 36984 | 23.584 | 0.145 | nm |
| Leucine | Leucine | Amino acids | 36963 | 0.074 | 0.023 | mmol/l |
| MUFA % | Ratio of monounsaturated fatty acids to total fatty acids | Fatty acids | 36855 | 26.914 | 2.963 | % |
| Omega-3 % | Ratio of omega-3 fatty acids to total fatty acids | Fatty acids | 36852 | 4.398 | 1.196 | % |
| Omega-6 % | Ratio of omega-6 fatty acids to total fatty acids | Fatty acids | 36855 | 32.843 | 3.398 | % |
| Phenylalanine | Phenylalanine | Amino acids | 36978 | 0.072 | 0.014 | mmol/l |
| Phosphatidylcholines | Phosphatidylcholines | Glycerides and phospholipids | 36902 | 1.925 | 0.409 | mmol/l |
| Phosphoglycerides | Phosphoglycerides | Glycerides and phospholipids | 36902 | 1.909 | 0.426 | mmol/l |
| PUFA % | Ratio of polyunsaturated fatty acids to total fatty acids | Fatty acids | 36855 | 37.240 | 3.679 | % |
| Pyruvate | Pyruvate | Glycolysis related metabolites | 36862 | 0.081 | 0.027 | mmol/l |
| Remnant-C | Remnant cholesterol (non-HDL, non-LDL -cholesterol) | Cholesterol | 36984 | 1.428 | 0.511 | mmol/l |
| SFA % | Ratio of saturated fatty acids to total fatty acids | Fatty acids | 36855 | 35.850 | 1.933 | % |
| Sphingomyelins | Sphingomyelins | Glycerides and phospholipids | 36894 | 0.453 | 0.107 | mmol/l |
| TG/PG | Ratio of triglycerides to phosphoglycerides | Glycerides and phospholipids | 36884 | 0.607 | 0.302 | ratio |
| Total cholesterol | Total cholesterol | Cholesterol | 36984 | 4.615 | 1.099 | mmol/l |
| Total cholines | Total cholines | Glycerides and phospholipids | 36902 | 2.302 | 0.458 | mmol/l |
| Total fatty acids | Total fatty acids | Fatty acids | 36855 | 11.389 | 2.848 | mmol/l |
| Total free cholesterol | Total free cholesterol | Cholesterol | 36905 | 1.361 | 0.312 | mmol/l |
| Total triglycerides | Total triglycerides | Glycerides and phospholipids | 36984 | 1.318 | 0.747 | mmol/l |
| Triglycerides in HDL | Triglycerides in HDL | Glycerides and phospholipids | 36984 | 0.151 | 0.050 | mmol/l |
| Triglycerides in LDL | Triglycerides in LDL | Glycerides and phospholipids | 36983 | 0.196 | 0.085 | mmol/l |
| Triglycerides in VLDL | Triglycerides in VLDL | Glycerides and phospholipids | 36984 | 0.845 | 0.651 | mmol/l |
| Tyrosine | Tyrosine | Amino acids | 36971 | 0.053 | 0.013 | mmol/l |
| Valine | Valine | Amino acids | 36902 | 0.170 | 0.045 | mmol/l |
| VLDL cholesterol | VLDL cholesterol | Cholesterol | 36984 | 0.709 | 0.338 | mmol/l |
| VLDL size | Average diameter for VLDL particles | Lipoprotein particle sizes | 36984 | 36.271 | 1.581 | nm |

VLDL, very low density lipoprotein; LDL, low density lipoprotein; HDL, high density lipoprotein.

**Table S2.** Description of lipoprotein subclasses.

| Abbreviation | Description | Diameter |
| --- | --- | --- |
| XXL-VLDL | Chylomicrons and extremely large VLDL particles | >75 nm |
| XL-VLDL | Very large VLDL particles | 64 nm |
| L-VLDL | Large VLDL particles | 53.6 nm |
| M-VLDL | Medium VLDL particles | 44.5 nm |
| S-VLDL | Small VLDL particles | 36.8 nm |
| XS-VLDL | Very small VLDL particles | 31.3 nm |
| IDL | IDL particles | 28.6 nm |
| L-LDL | Large LDL particles | 25.5 nm |
| M-LDL | Medium LDL particles | 23.0 nm |
| S-LDL | Small LDL particles | 18.7 nm |
| XL-HDL | Very large HDL particles | 14.3 nm |
| L-HDL | Large HDL particles | 12.1 nm |
| M-HDL | Medium HDL particles | 10.9 nm |
| S-HDL | Small HDL particles | 8.7 nm |

The lipoprotein particle subclasses are expressed in the units of mol/l or mmol/l.

**Table S3.** Characteristics of the longitudinal study sample.

|  | FINRISK | | Health | |
| --- | --- | --- | --- | --- |
| Characteristics | 2007 | 2014 | 2000 | 2011 |
| N | 968 | 968 | 3229 | 3229 |
| Age, y (SD) | 53.0 (12.7) | 58.0 (12.7) | 48.3 (11.3) | 59.3 (11.3) |
| Female, N (%) | 544 (56.2) | 544 (56.2) | 1779 (55.1) | 1779 (55.1) |
| BMI, kg/m² (SD) | 26.7 (4.5) | 26.6 (4.6) | 26.5 (4.3) | 27.3 (4.8) |
| Systolic BP, mmHg (SD) | 133.2 (18.4) | 125.8 (19.6) | 130.7 (19.3) | 134.2 (18.2) |
| Diastolic BP, mmHg (SD) | 78.6 (10.5) | 81.4 (10.4) | 81.5 (10.7) | 80.5 (10.1) |
| Hypertension, N (%) | 433 (44.7) | 445 (46.0) | 1231 (38.1) | 1733 (53.7) |
| Current smoker, N (%) | 155 (16.0) | 80 (8.3) | 613 (19.0) | 437 (13.5) |
| Diabetes mellitus, N (%) | 34 (3.5) | 89 (9.2) | 95 (2.9) | 284 (8.8) |
| Exercise, N (%) |  |  |  |  |
| Light | 178 (18.4) | 182 (18.8) | 703 (21.8) | 812 (25.1) |
| Moderate | 504 (52.1) | 537 (55.5) | 1836 (56.9) | 1711 (53.0) |
| Heavy | 275 (28.4) | 240 (24.8) | 639 (19.8) | 658 (20.4) |
| Competitive | 11 (1.1) | 9 (0.9) | 51 (1.6) | 48 (1.5) |
| Antihypertensive  medication, N (%) | 193 (19.9) | 300 (31.0) | 405 (12.5) | 952 (29.5) |
| Lipid medication, N (%) | 125 (12.9) | 191 (19.7) | 161 (5.0) | 596 (18.5) |

Continuous variables are presented as mean (standard deviation) and categorical values as count (percent). BP indicates blood pressure, BMI, body mass index.

**Table S4.** Association between metabolic measures and blood pressure indices in the cross-sectional sample.

|  | Systolic BP | | Diastolic BP | | Hypertension | |
| --- | --- | --- | --- | --- | --- | --- |
| Metabolite | β (95% CI) | p | β (95% CI) | p | OR (95% CI) | p |
| Acetate | -0.28 (-0.46 to -0.11) | 0.002 | 0.13 (0.02 to 0.24) | 0.02 | 0.97 (0.95 to 1.00) | 0.02 |
| Acetoacetate | 0.57 (0.39 to 0.76) | <0.001 | 0.34 (0.23 to 0.45) | <0.001 | 1.06 (1.04 to 1.09) | <0.001 |
| Alanine | 0.58 (0.36 to 0.80) | <0.001 | 0.50 (0.37 to 0.64) | <0.001 | 1.14 (1.10 to 1.17) | <0.001 |
| Albumin | 1.84 (1.64 to 2.03) | <0.001 | 1.02 (0.91 to 1.14) | <0.001 | 1.24 (1.20 to 1.27) | <0.001 |
| ApoB/ApoA1 | 0.19 (-0.01 to 0.40) | 0.07 | 0.96 (0.84 to 1.08) | <0.001 | 1.10 (1.07 to 1.13) | <0.001 |
| Apolipoprotein A1 | 1.52 (1.33 to 1.70) | <0.001 | 0.92 (0.81 to 1.04) | <0.001 | 1.10 (1.07 to 1.13) | <0.001 |
| Apolipoprotein B | 1.02 (0.82 to 1.21) | <0.001 | 1.45 (1.33 to 1.57) | <0.001 | 1.15 (1.12 to 1.18) | <0.001 |
| Beta-hydroxybutyrate | 1.21 (1.03 to 1.39) | <0.001 | 0.38 (0.28 to 0.49) | <0.001 | 1.11 (1.08 to 1.14) | <0.001 |
| Citrate | 0.31 (0.12 to 0.49) | 0.001 | -0.28 (-0.40 to -0.17) | <0.001 | 1.00 (0.98 to 1.03) | 0.81 |
| Creatinine | -0.58 (-0.78 to -0.38) | <0.001 | -0.12 (-0.25 to -0.00) | 0.05 | 1.00 (0.97 to 1.03) | 0.88 |
| DHA % | -0.25 (-0.43 to -0.06) | 0.01 | -0.07 (-0.19 to 0.04) | 0.23 | 0.98 (0.96 to 1.01) | 0.14 |
| Degree of unsaturation | -0.84 (-1.03 to -0.65) | <0.001 | -0.63 (-0.75 to -0.51) | <0.001 | 0.87 (0.84 to 0.89) | <0.001 |
| Esterified-C | 1.32 (1.13 to 1.52) | <0.001 | 1.37 (1.25 to 1.49) | <0.001 | 1.10 (1.08 to 1.13) | <0.001 |
| Glucose | 1.16 (0.96 to 1.36) | <0.001 | 0.44 (0.32 to 0.56) | <0.001 | 1.17 (1.13 to 1.21) | <0.001 |
| Glutamine | -0.45 (-0.66 to -0.24) | <0.001 | -0.13 (-0.26 to -0.01) | 0.04 | 0.91 (0.89 to 0.94) | <0.001 |
| Glycerol | 2.13 (1.88 to 2.38) | <0.001 | 1.10 (0.94 to 1.25) | <0.001 | 1.34 (1.29 to 1.39) | <0.001 |
| Glycine | -0.51 (-0.73 to -0.29) | <0.001 | 0.09 (-0.04 to 0.22) | 0.19 | 0.95 (0.92 to 0.97) | <0.001 |
| Glycoprotein acetyls | 1.25 (1.05 to 1.45) | <0.001 | 1.03 (0.90 to 1.15) | <0.001 | 1.28 (1.24 to 1.32) | <0.001 |
| HDL cholesterol | 1.26 (1.06 to 1.47) | <0.001 | 0.39 (0.27 to 0.52) | <0.001 | 1.03 (1.01 to 1.06) | 0.02 |
| HDL size | 0.17 (-0.05 to 0.39) | 0.14 | -0.60 (-0.73 to -0.46) | <0.001 | 0.88 (0.85 to 0.91) | <0.001 |
| HDL2 cholesterol | 1.20 (1.00 to 1.40) | <0.001 | 0.35 (0.23 to 0.48) | <0.001 | 1.03 (1.00 to 1.06) | 0.03 |
| HDL3 cholesterol | 1.55 (1.33 to 1.77) | <0.001 | 0.81 (0.68 to 0.94) | <0.001 | 1.10 (1.07 to 1.13) | <0.001 |
| Histidine | -0.06 (-0.27 to 0.15) | 0.57 | 0.06 (-0.07 to 0.19) | 0.36 | 0.99 (0.97 to 1.02) | 0.73 |
| Isoleucine | 0.47 (0.28 to 0.67) | <0.001 | 0.55 (0.43 to 0.66) | <0.001 | 1.09 (1.06 to 1.12) | <0.001 |
| LA % | -1.44 (-1.65 to -1.23) | <0.001 | -0.94 (-1.06 to -0.81) | <0.001 | 0.78 (0.76 to 0.80) | <0.001 |
| LDL cholesterol | 0.79 (0.60 to 0.98) | <0.001 | 1.14 (1.02 to 1.25) | <0.001 | 1.06 (1.03 to 1.08) | <0.001 |
| LDL size | -0.25 (-0.46 to -0.04) | 0.02 | -0.91 (-1.04 to -0.78) | <0.001 | 0.91 (0.88 to 0.93) | <0.001 |
| Lactate | 1.49 (1.30 to 1.68) | <0.001 | 0.62 (0.50 to 0.74) | <0.001 | 1.28 (1.24 to 1.32) | <0.001 |
| Leucine | 0.40 (0.19 to 0.61) | <0.001 | 0.60 (0.47 to 0.72) | <0.001 | 1.09 (1.06 to 1.12) | <0.001 |
| MUFA % | 1.17 (0.97 to 1.36) | <0.001 | 1.01 (0.89 to 1.13) | <0.001 | 1.25 (1.21 to 1.28) | <0.001 |
| Omega-3 % | -0.30 (-0.49 to -0.12) | 0.001 | -0.05 (-0.16 to 0.06) | 0.38 | 0.97 (0.95 to 1.00) | 0.04 |
| Omega-6 % | -1.32 (-1.52 to -1.12) | <0.001 | -0.97 (-1.09 to -0.85) | <0.001 | 0.78 (0.76 to 0.81) | <0.001 |
| PUFA % | -1.27 (-1.47 to -1.07) | <0.001 | -0.87 (-0.99 to -0.75) | <0.001 | 0.80 (0.78 to 0.82) | <0.001 |
| Phenylalanine | 0.25 (0.00 to 0.50) | 0.05 | 0.38 (0.23 to 0.53) | <0.001 | 1.10 (1.06 to 1.14) | <0.001 |
| Phosphatidylcholines | 1.75 (1.55 to 1.95) | <0.001 | 1.42 (1.30 to 1.54) | <0.001 | 1.18 (1.15 to 1.21) | <0.001 |
| Phosphoglycerides | 1.76 (1.56 to 1.95) | <0.001 | 1.45 (1.33 to 1.57) | <0.001 | 1.19 (1.16 to 1.22) | <0.001 |
| Pyruvate | 0.93 (0.74 to 1.12) | <0.001 | 0.21 (0.09 to 0.32) | <0.001 | 1.23 (1.19 to 1.26) | <0.001 |
| Remnant-C | 1.13 (0.92 to 1.34) | <0.001 | 1.46 (1.34 to 1.59) | <0.001 | 1.15 (1.12 to 1.18) | <0.001 |
| SFA % | 0.71 (0.51 to 0.92) | <0.001 | 0.13 (0.01 to 0.26) | 0.04 | 1.10 (1.07 to 1.14) | <0.001 |
| Sphingomyelins | 1.41 (1.18 to 1.64) | <0.001 | 1.22 (1.08 to 1.36) | <0.001 | 1.09 (1.05 to 1.12) | <0.001 |
| TG/PG | 0.17 (-0.03 to 0.36) | 0.10 | 0.68 (0.56 to 0.80) | <0.001 | 1.16 (1.12 to 1.19) | <0.001 |
| Total cholesterol | 1.32 (1.13 to 1.52) | <0.001 | 1.35 (1.23 to 1.47) | <0.001 | 1.10 (1.07 to 1.13) | <0.001 |
| Total cholines | 1.69 (1.49 to 1.90) | <0.001 | 1.40 (1.27 to 1.53) | <0.001 | 1.16 (1.13 to 1.19) | <0.001 |
| Total fatty acids | 1.53 (1.33 to 1.72) | <0.001 | 1.57 (1.45 to 1.68) | <0.001 | 1.22 (1.19 to 1.25) | <0.001 |
| Total free cholesterol | 1.29 (1.09 to 1.48) | <0.001 | 1.27 (1.15 to 1.38) | <0.001 | 1.10 (1.07 to 1.13) | <0.001 |
| Total triglycerides | 0.96 (0.76 to 1.15) | <0.001 | 1.18 (1.06 to 1.29) | <0.001 | 1.22 (1.18 to 1.25) | <0.001 |
| Triglycerides in HDL | 1.33 (1.15 to 1.52) | <0.001 | 1.00 (0.89 to 1.12) | <0.001 | 1.19 (1.16 to 1.22) | <0.001 |
| Triglycerides in LDL | 1.45 (1.25 to 1.65) | <0.001 | 1.03 (0.90 to 1.15) | <0.001 | 1.18 (1.15 to 1.22) | <0.001 |
| Triglycerides in VLDL | 0.75 (0.56 to 0.94) | <0.001 | 1.11 (0.99 to 1.22) | <0.001 | 1.20 (1.17 to 1.24) | <0.001 |
| Tyrosine | -0.05 (-0.23 to 0.14) | 0.61 | 0.18 (0.07 to 0.30) | 0.002 | 1.02 (1.00 to 1.05) | 0.09 |
| VLDL cholesterol | 1.00 (0.79 to 1.21) | <0.001 | 1.38 (1.26 to 1.51) | <0.001 | 1.18 (1.15 to 1.22) | <0.001 |
| VLDL size | 0.38 (0.17 to 0.59) | <0.001 | 0.84 (0.72 to 0.97) | <0.001 | 1.17 (1.14 to 1.21) | <0.001 |
| Valine | -0.11 (-0.32 to 0.10) | 0.32 | 0.05 (-0.08 to 0.18) | 0.48 | 0.98 (0.95 to 1.01) | 0.24 |

**Table S5.** Cross-sectional associations between metabolic measures and hypertension by sex.

|  | Male | | Female | |
| --- | --- | --- | --- | --- |
| Metabolite | OR (95% CI) | p | OR (95% CI) | p |
| Acetate | -0.00 (-0.03 to 0.02) | 0.87 | -0.13 (-0.21 to -0.06) | <0.001 |
| Acetoacetate | 0.09 (0.06 to 0.12) | <0.001 | 0.02 (-0.02 to 0.06) | 0.32 |
| Alanine | 0.14 (0.09 to 0.18) | <0.001 | 0.11 (0.06 to 0.15) | <0.001 |
| Albumin | 0.24 (0.20 to 0.28) | <0.001 | 0.16 (0.12 to 0.20) | <0.001 |
| ApoB/ApoA1 | 0.08 (0.04 to 0.11) | <0.001 | 0.06 (0.02 to 0.10) | 0.009 |
| Apolipoprotein A1 | 0.14 (0.11 to 0.18) | <0.001 | 0.05 (0.01 to 0.08) | 0.01 |
| Apolipoprotein B | 0.14 (0.10 to 0.17) | <0.001 | 0.08 (0.04 to 0.12) | <0.001 |
| Beta-hydroxybutyrate | 0.14 (0.10 to 0.17) | <0.001 | 0.09 (0.05 to 0.13) | <0.001 |
| Citrate | 0.01 (-0.03 to 0.05) | 0.65 | 0.01 (-0.03 to 0.05) | 0.62 |
| Creatinine | -0.01 (-0.05 to 0.02) | 0.45 | -0.01 (-0.06 to 0.04) | 0.70 |
| DHA % | 0.00 (-0.03 to 0.04) | 0.98 | -0.05 (-0.08 to -0.01) | 0.02 |
| Degree of unsaturation | -0.14 (-0.17 to -0.10) | <0.001 | -0.14 (-0.18 to -0.10) | <0.001 |
| Esterified-C | 0.10 (0.06 to 0.14) | <0.001 | 0.05 (0.02 to 0.09) | 0.010 |
| Glucose | 0.15 (0.11 to 0.20) | <0.001 | 0.13 (0.08 to 0.18) | <0.001 |
| Glutamine | -0.16 (-0.20 to -0.12) | <0.001 | -0.06 (-0.11 to -0.02) | 0.004 |
| Glycerol | 0.31 (0.25 to 0.36) | <0.001 | 0.25 (0.20 to 0.31) | <0.001 |
| Glycine | -0.05 (-0.10 to 0.01) | 0.15 | -0.08 (-0.12 to -0.05) | <0.001 |
| Glycoprotein acetyls | 0.24 (0.20 to 0.28) | <0.001 | 0.23 (0.18 to 0.27) | <0.001 |
| HDL cholesterol | 0.06 (0.02 to 0.10) | 0.008 | 0.02 (-0.02 to 0.06) | 0.38 |
| HDL size | -0.10 (-0.15 to -0.06) | <0.001 | -0.13 (-0.17 to -0.09) | <0.001 |
| HDL2 cholesterol | 0.06 (0.02 to 0.10) | 0.003 | 0.01 (-0.03 to 0.05) | 0.58 |
| HDL3 cholesterol | 0.08 (0.04 to 0.12) | <0.001 | 0.09 (0.05 to 0.13) | <0.001 |
| Histidine | -0.01 (-0.05 to 0.03) | 0.65 | -0.02 (-0.06 to 0.03) | 0.50 |
| Isoleucine | 0.07 (0.04 to 0.11) | <0.001 | 0.07 (0.03 to 0.11) | 0.002 |
| LA % | -0.27 (-0.31 to -0.23) | <0.001 | -0.20 (-0.24 to -0.15) | <0.001 |
| LDL cholesterol | 0.04 (0.01 to 0.08) | 0.02 | 0.03 (-0.01 to 0.07) | 0.20 |
| LDL size | -0.08 (-0.12 to -0.04) | <0.001 | -0.07 (-0.12 to -0.03) | 0.004 |
| Lactate | 0.24 (0.21 to 0.28) | <0.001 | 0.25 (0.21 to 0.30) | <0.001 |
| Leucine | 0.07 (0.04 to 0.11) | <0.001 | 0.06 (0.01 to 0.10) | 0.02 |
| MUFA % | 0.20 (0.16 to 0.24) | <0.001 | 0.21 (0.17 to 0.25) | <0.001 |
| Omega-3 % | -0.01 (-0.04 to 0.03) | 0.66 | -0.06 (-0.10 to -0.02) | 0.002 |
| Omega-6 % | -0.23 (-0.27 to -0.20) | <0.001 | -0.22 (-0.26 to -0.18) | <0.001 |
| PUFA % | -0.21 (-0.24 to -0.17) | <0.001 | -0.22 (-0.26 to -0.18) | <0.001 |
| Phenylalanine | 0.14 (0.09 to 0.19) | <0.001 | 0.06 (0.01 to 0.11) | 0.02 |
| Phosphatidylcholines | 0.21 (0.17 to 0.25) | <0.001 | 0.09 (0.05 to 0.13) | <0.001 |
| Phosphoglycerides | 0.21 (0.18 to 0.25) | <0.001 | 0.10 (0.06 to 0.14) | <0.001 |
| Pyruvate | 0.23 (0.19 to 0.27) | <0.001 | 0.19 (0.15 to 0.23) | <0.001 |
| Remnant-C | 0.14 (0.10 to 0.18) | <0.001 | 0.08 (0.04 to 0.13) | <0.001 |
| SFA % | 0.11 (0.07 to 0.15) | <0.001 | 0.09 (0.05 to 0.14) | <0.001 |
| Sphingomyelins | 0.10 (0.05 to 0.14) | <0.001 | 0.03 (-0.02 to 0.08) | 0.23 |
| TG/PG | 0.12 (0.09 to 0.16) | <0.001 | 0.13 (0.08 to 0.18) | <0.001 |
| Total cholesterol | 0.10 (0.06 to 0.14) | <0.001 | 0.05 (0.01 to 0.09) | 0.01 |
| Total cholines | 0.19 (0.14 to 0.23) | <0.001 | 0.08 (0.04 to 0.12) | <0.001 |
| Total fatty acids | 0.20 (0.17 to 0.24) | <0.001 | 0.14 (0.10 to 0.18) | <0.001 |
| Total free cholesterol | 0.09 (0.06 to 0.13) | <0.001 | 0.05 (0.01 to 0.09) | 0.01 |
| Total triglycerides | 0.17 (0.14 to 0.21) | <0.001 | 0.17 (0.13 to 0.22) | <0.001 |
| Triglycerides in HDL | 0.20 (0.17 to 0.24) | <0.001 | 0.12 (0.09 to 0.16) | <0.001 |
| Triglycerides in LDL | 0.17 (0.13 to 0.21) | <0.001 | 0.13 (0.09 to 0.18) | <0.001 |
| Triglycerides in VLDL | 0.16 (0.13 to 0.19) | <0.001 | 0.17 (0.12 to 0.22) | <0.001 |
| Tyrosine | 0.02 (-0.02 to 0.05) | 0.44 | -0.00 (-0.04 to 0.04) | 0.97 |
| VLDL cholesterol | 0.16 (0.12 to 0.20) | <0.001 | 0.12 (0.07 to 0.16) | <0.001 |
| VLDL size | 0.15 (0.11 to 0.18) | <0.001 | 0.14 (0.09 to 0.18) | <0.001 |
| Valine | -0.05 (-0.09 to -0.01) | 0.02 | -0.01 (-0.05 to 0.04) | 0.79 |

**Table S6.** Cross-sectional associations between metabolic measures and hypertension in the younger and older participants.

|  | Younger than median age | | Older than median age | |
| --- | --- | --- | --- | --- |
| Metabolite | OR (95% CI) | p | OR (95% CI) | p |
| Acetate | -0.07 (-0.12 to -0.02) | 0.006 | -0.01 (-0.04 to 0.02) | 0.44 |
| Acetoacetate | 0.06 (0.03 to 0.10) | <0.001 | 0.05 (0.02 to 0.09) | 0.004 |
| Alanine | 0.15 (0.10 to 0.19) | <0.001 | 0.11 (0.07 to 0.15) | <0.001 |
| Albumin | 0.20 (0.16 to 0.25) | <0.001 | 0.20 (0.16 to 0.24) | <0.001 |
| ApoB/ApoA1 | 0.11 (0.07 to 0.15) | <0.001 | 0.05 (0.01 to 0.09) | 0.02 |
| Apolipoprotein A1 | 0.12 (0.08 to 0.16) | <0.001 | 0.06 (0.02 to 0.09) | 0.003 |
| Apolipoprotein B | 0.16 (0.12 to 0.20) | <0.001 | 0.08 (0.04 to 0.11) | <0.001 |
| Beta-hydroxybutyrate | 0.10 (0.07 to 0.14) | <0.001 | 0.11 (0.08 to 0.15) | <0.001 |
| Citrate | 0.02 (-0.02 to 0.06) | 0.28 | 0.00 (-0.03 to 0.04) | 0.83 |
| Creatinine | -0.00 (-0.05 to 0.04) | 0.86 | -0.00 (-0.04 to 0.03) | 0.83 |
| DHA % | -0.04 (-0.08 to 0.00) | 0.09 | -0.02 (-0.05 to 0.02) | 0.43 |
| Degree of unsaturation | -0.19 (-0.23 to -0.15) | <0.001 | -0.11 (-0.14 to -0.08) | <0.001 |
| Esterified-C | 0.12 (0.07 to 0.16) | <0.001 | 0.04 (0.00 to 0.08) | 0.05 |
| Glucose | 0.18 (0.13 to 0.23) | <0.001 | 0.12 (0.08 to 0.17) | <0.001 |
| Glutamine | -0.13 (-0.17 to -0.08) | <0.001 | -0.10 (-0.14 to -0.06) | <0.001 |
| Glycerol | 0.30 (0.24 to 0.35) | <0.001 | 0.27 (0.22 to 0.32) | <0.001 |
| Glycine | -0.04 (-0.09 to 0.01) | 0.12 | -0.09 (-0.13 to -0.05) | <0.001 |
| Glycoprotein acetyls | 0.25 (0.21 to 0.29) | <0.001 | 0.22 (0.18 to 0.26) | <0.001 |
| HDL cholesterol | 0.05 (0.00 to 0.09) | 0.04 | 0.01 (-0.02 to 0.05) | 0.60 |
| HDL size | -0.11 (-0.16 to -0.06) | <0.001 | -0.13 (-0.17 to -0.09) | <0.001 |
| HDL2 cholesterol | 0.04 (0.00 to 0.09) | 0.05 | 0.01 (-0.02 to 0.05) | 0.54 |
| HDL3 cholesterol | 0.09 (0.05 to 0.14) | <0.001 | 0.06 (0.02 to 0.10) | 0.006 |
| Histidine | -0.02 (-0.06 to 0.02) | 0.40 | -0.01 (-0.05 to 0.03) | 0.73 |
| Isoleucine | 0.09 (0.05 to 0.13) | <0.001 | 0.07 (0.03 to 0.10) | <0.001 |
| LA % | -0.29 (-0.33 to -0.24) | <0.001 | -0.19 (-0.23 to -0.15) | <0.001 |
| LDL cholesterol | 0.06 (0.02 to 0.10) | 0.004 | 0.01 (-0.03 to 0.04) | 0.74 |
| LDL size | -0.09 (-0.14 to -0.05) | <0.001 | -0.06 (-0.11 to -0.02) | 0.004 |
| Lactate | 0.28 (0.24 to 0.32) | <0.001 | 0.23 (0.19 to 0.27) | <0.001 |
| Leucine | 0.08 (0.04 to 0.12) | <0.001 | 0.06 (0.02 to 0.10) | 0.003 |
| MUFA % | 0.25 (0.21 to 0.29) | <0.001 | 0.17 (0.13 to 0.20) | <0.001 |
| Omega-3 % | -0.04 (-0.09 to -0.00) | 0.05 | -0.03 (-0.06 to 0.00) | 0.11 |
| Omega-6 % | -0.27 (-0.31 to -0.23) | <0.001 | -0.19 (-0.23 to -0.15) | <0.001 |
| PUFA % | -0.26 (-0.30 to -0.22) | <0.001 | -0.17 (-0.21 to -0.14) | <0.001 |
| Phenylalanine | 0.14 (0.09 to 0.20) | <0.001 | 0.07 (0.02 to 0.11) | 0.007 |
| Phosphatidylcholines | 0.19 (0.15 to 0.23) | <0.001 | 0.09 (0.06 to 0.13) | <0.001 |
| Phosphoglycerides | 0.20 (0.16 to 0.24) | <0.001 | 0.11 (0.07 to 0.14) | <0.001 |
| Pyruvate | 0.24 (0.20 to 0.28) | <0.001 | 0.19 (0.15 to 0.23) | <0.001 |
| Remnant-C | 0.16 (0.12 to 0.20) | <0.001 | 0.07 (0.04 to 0.11) | <0.001 |
| SFA % | 0.12 (0.08 to 0.16) | <0.001 | 0.09 (0.05 to 0.13) | <0.001 |
| Sphingomyelins | 0.10 (0.06 to 0.15) | <0.001 | 0.02 (-0.02 to 0.06) | 0.48 |
| TG/PG | 0.14 (0.10 to 0.18) | <0.001 | 0.12 (0.08 to 0.16) | <0.001 |
| Total cholesterol | 0.12 (0.07 to 0.16) | <0.001 | 0.04 (0.00 to 0.07) | 0.06 |
| Total cholines | 0.18 (0.13 to 0.22) | <0.001 | 0.08 (0.04 to 0.12) | <0.001 |
| Total fatty acids | 0.22 (0.18 to 0.26) | <0.001 | 0.13 (0.09 to 0.17) | <0.001 |
| Total free cholesterol | 0.11 (0.07 to 0.15) | <0.001 | 0.03 (-0.00 to 0.07) | 0.10 |
| Total triglycerides | 0.20 (0.16 to 0.24) | <0.001 | 0.15 (0.11 to 0.19) | <0.001 |
| Triglycerides in HDL | 0.18 (0.14 to 0.22) | <0.001 | 0.15 (0.11 to 0.18) | <0.001 |
| Triglycerides in LDL | 0.18 (0.14 to 0.22) | <0.001 | 0.12 (0.08 to 0.16) | <0.001 |
| Triglycerides in VLDL | 0.19 (0.15 to 0.23) | <0.001 | 0.15 (0.11 to 0.19) | <0.001 |
| Tyrosine | 0.03 (-0.01 to 0.07) | 0.10 | 0.00 (-0.03 to 0.03) | 0.98 |
| VLDL cholesterol | 0.18 (0.14 to 0.23) | <0.001 | 0.11 (0.07 to 0.15) | <0.001 |
| VLDL size | 0.16 (0.12 to 0.20) | <0.001 | 0.13 (0.10 to 0.17) | <0.001 |
| Valine | -0.04 (-0.08 to 0.00) | 0.09 | -0.01 (-0.05 to 0.03) | 0.54 |

**Table S7.** The association between baseline metabolite levels and systolic BP change between baseline and follow-up.

| Metabolite | β (95% CI) | p |
| --- | --- | --- |
| Acetate | 0.83 (0.25 to 1.41) | 0.02 |
| Acetoacetate | -0.22 (-0.73 to 0.29) | 0.62 |
| Alanine | -0.06 (-0.67 to 0.54) | 0.87 |
| Albumin | 0.26 (-0.16 to 0.67) | 0.40 |
| ApoB/ApoA1 | 0.77 (0.23 to 1.31) | 0.02 |
| Apolipoprotein A1 | -0.15 (-0.62 to 0.32) | 0.73 |
| Apolipoprotein B | 0.63 (0.14 to 1.11) | 0.03 |
| Beta-hydroxybutyrate | 0.11 (-0.38 to 0.60) | 0.76 |
| Citrate | -0.28 (-0.84 to 0.28) | 0.54 |
| Creatinine | 0.45 (-0.13 to 1.03) | 0.26 |
| DHA % | 0.26 (-0.21 to 0.74) | 0.48 |
| Degree of unsaturation | 0.33 (-0.18 to 0.84) | 0.38 |
| Esterified-C | 0.47 (0.03 to 0.92) | 0.09 |
| Glucose | 0.58 (0.02 to 1.14) | 0.10 |
| Glutamine | 0.29 (-0.29 to 0.88) | 0.54 |
| Glycerol | 0.08 (-0.60 to 0.77) | 0.84 |
| Glycine | -0.05 (-0.54 to 0.44) | 0.87 |
| Glycoprotein acetyls | 0.50 (-0.02 to 1.02) | 0.13 |
| HDL cholesterol | -0.39 (-0.92 to 0.13) | 0.28 |
| HDL size | -0.89 (-1.46 to -0.32) | 0.01 |
| HDL2 cholesterol | -0.47 (-1.00 to 0.06) | 0.17 |
| HDL3 cholesterol | 0.20 (-0.29 to 0.69) | 0.65 |
| Histidine | 0.19 (-0.35 to 0.72) | 0.72 |
| Isoleucine (branched) | 0.09 (-0.45 to 0.62) | 0.80 |
| LA % | -0.42 (-1.09 to 0.25) | 0.39 |
| LDL cholesterol | 0.74 (0.28 to 1.20) | 0.010 |
| LDL size | -0.63 (-1.20 to -0.05) | 0.08 |
| Lactate | 0.28 (-0.35 to 0.91) | 0.62 |
| Leucine (branched) | 0.14 (-0.40 to 0.67) | 0.76 |
| MUFA % | 0.19 (-0.35 to 0.74) | 0.71 |
| Omega-3 % | 0.32 (-0.15 to 0.80) | 0.34 |
| Omega-6 % | -0.17 (-0.81 to 0.47) | 0.76 |
| PUFA % | 0.03 (-0.57 to 0.62) | 0.94 |
| Phenylalanine (aromatic) | 0.11 (-0.45 to 0.68) | 0.77 |
| Phosphatidylcholines | 0.14 (-0.31 to 0.59) | 0.74 |
| Phosphoglycerides | 0.17 (-0.28 to 0.62) | 0.69 |
| Pyruvate | 0.15 (-0.44 to 0.74) | 0.76 |
| Remnant-C | 0.62 (0.14 to 1.10) | 0.03 |
| SFA % | -0.45 (-1.08 to 0.18) | 0.31 |
| Sphingomyelins | 0.49 (-0.02 to 1.01) | 0.13 |
| TG/PG | 0.13 (-0.43 to 0.69) | 0.76 |
| Total cholesterol | 0.48 (0.03 to 0.92) | 0.09 |
| Total cholines | 0.24 (-0.25 to 0.73) | 0.55 |
| Total fatty acids | 0.34 (-0.14 to 0.82) | 0.32 |
| Total free cholesterol | 0.49 (0.04 to 0.95) | 0.08 |
| Total triglycerides | 0.20 (-0.32 to 0.73) | 0.69 |
| Triglycerides in HDL | 0.05 (-0.44 to 0.53) | 0.87 |
| Triglycerides in LDL | 0.57 (0.06 to 1.09) | 0.07 |
| Triglycerides in VLDL | 0.13 (-0.40 to 0.66) | 0.76 |
| Tyrosine (aromatic) | -0.07 (-0.62 to 0.47) | 0.83 |
| VLDL cholesterol | 0.52 (0.01 to 1.02) | 0.10 |
| VLDL size | 0.10 (-0.44 to 0.64) | 0.78 |
| Valine (branched) | 0.25 (-0.27 to 0.78) | 0.56 |

**Table S8.** The gradient boosting method hyperparameters after Bayesian optimization.

| Sample | Covariates | Nrounds | Eta | Gamma | Max_depth | Min_child_weight | Subsample | Colsample_bytree |
| --- | --- | --- | --- | --- | --- | --- | --- | --- |
| Cross-sectional | Full model | 1530 | 0.0688948 | 8.765413 | 2 | 194 | 0.6513148 | 0.4494352 |
| Longitudinal | Full model | 3444 | 0.0050759 | 83.96593 | 4 | 158 | 0.4548142 | 0.4355926 |
| Cross-sectional | Metabolic measures | 516 | 0.035097 | 25.11328 | 5 | 99 | 0.6798628 | 0.7508606 |
| Longitudinal | Metabolic measures | 237 | 0.0513926 | 28.91418 | 3 | 142 | 0.297844 | 0.4839966 |
| Cross-sectional | Clinical characteristics | 4049 | 0.0010218 | 86.81831 | 7 | 125 | 0.2604274 | 0.6518759 |
| Longitudinal | Clinical characteristics | 1989 | 0.0829398 | 29.99735 | 2 | 188 | 0.447924 | 0.201896 |

We used gbtree booster, RMSE eval metrics and reg:squarederror objective in all models. Full model included both clinical characteristics and 53 circulating metabolic biomarkers. In models adjusted with clinical characteristics, we used following covariates age, sex, BMI, current smoking, diabetes, antihypertensive medication, exercise, and lipid medication, and baseline systolic BP (only in longitudinal model).

**Supplemental Figures**

**Figure S1.** Flow diagram for selection of cross-sectional and longitudinal samples.

The cross-sectional sample is combined from four independent FINRISK cohorts between 1997-2012 and HEALTH 2000. Longitudinal samples combined for the two cohorts with follow-up data: Health 2000-2011 and DILGOM.

**Figure S2.** Cross-sectional associations between the metabolites and hypertension (N=36985).


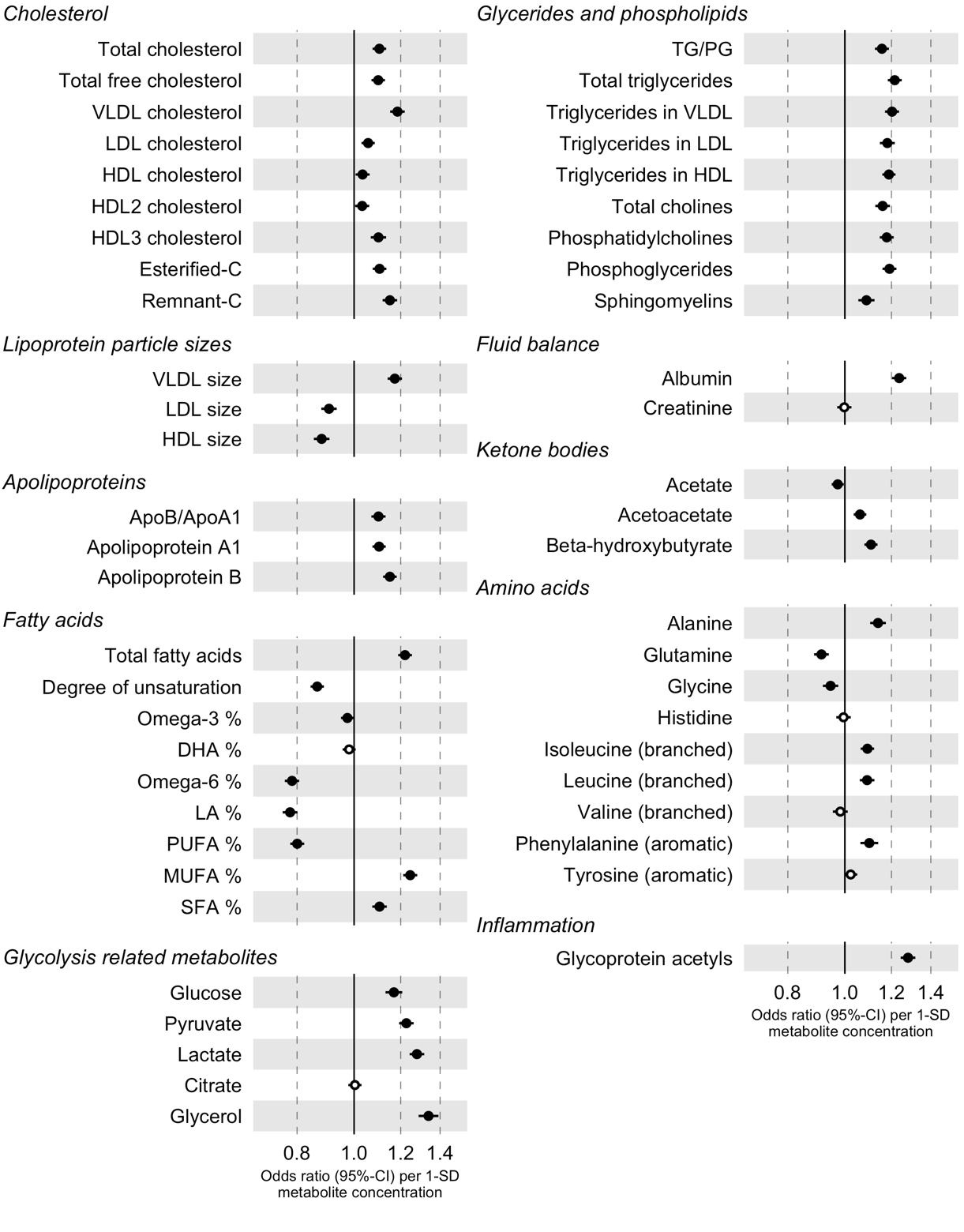


Filled circle signifies FDR-corrected *P*<0.05. Associations are adjusted for age, sex, BMI, current smoking, diabetes, exercise, lipid medication, and cohort. MUFA, monounsaturated fatty acids; SFA, saturated fatty acids; TG, triglycerides; PG, phosphoglycerides; VLDL, very low density lipoprotein; LDL, low density lipoprotein; HDL, high density lipoprotein Apo, apolipoprotein; C, cholesterol; DHA, docosahexaenoic acid; PUFA, polyunsaturated fatty acids; LA, linoleic acid.

**Figure S3.** Cross-sectional associations between metabolic measures and hypertension by sex.


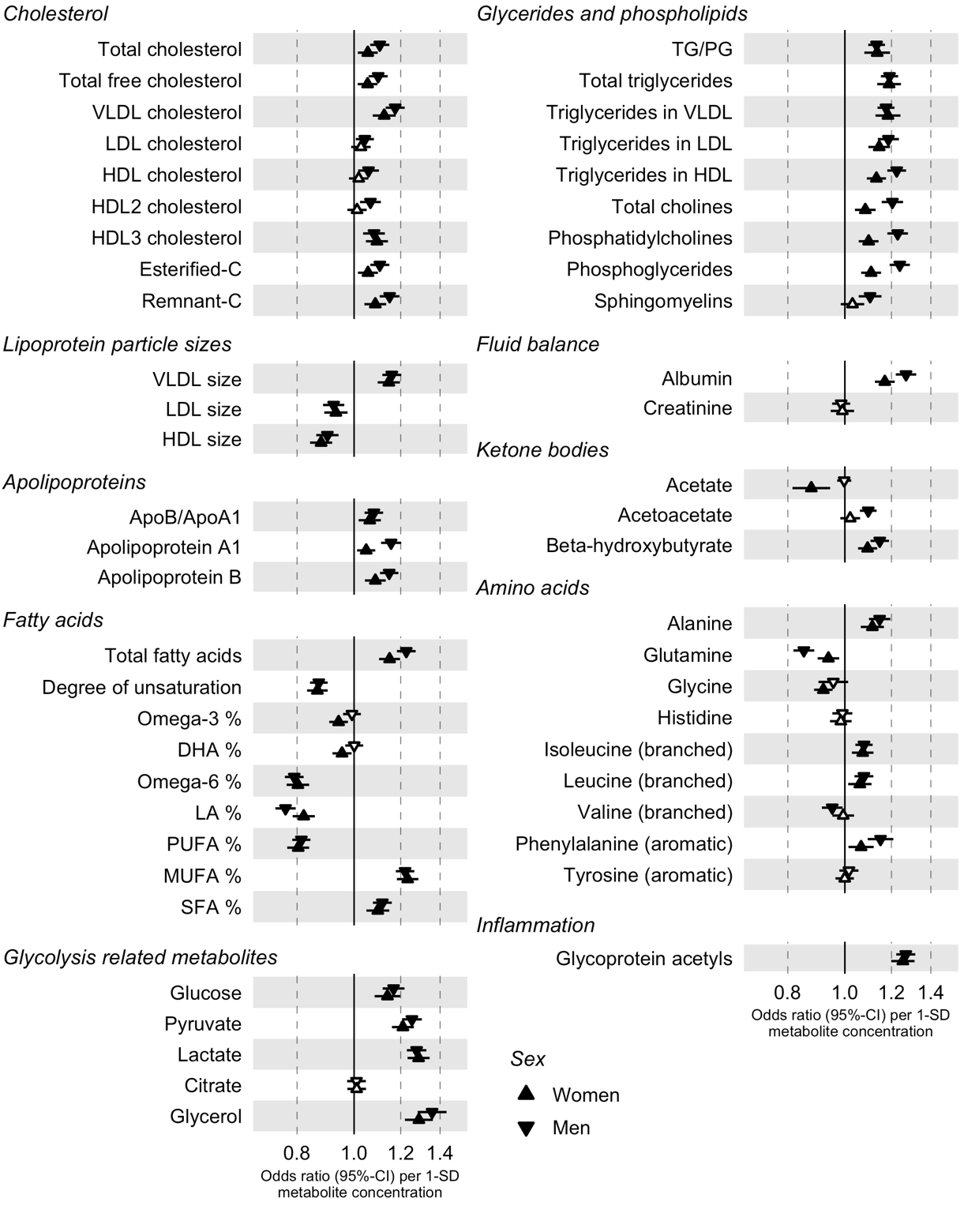


Filled triangle signifies FDR-corrected *P*<0.05. Associations are adjusted for age, sex, BMI, current smoking, diabetes, exercise, lipid medication, and cohort. MUFA, monounsaturated fatty acids; SFA, saturated fatty acids; TG, triglycerides; PG, phosphoglycerides; VLDL, very low density lipoprotein; LDL, low density lipoprotein; HDL, high density lipoprotein; Apo, apolipoprotein; C, cholesterol; DHA, docosahexaenoic acid; PUFA, polyunsaturated fatty acids; LA, linoleic acid.

**Figure S4.** Cross-sectional associations between metabolites and hypertension in younger and older participants.


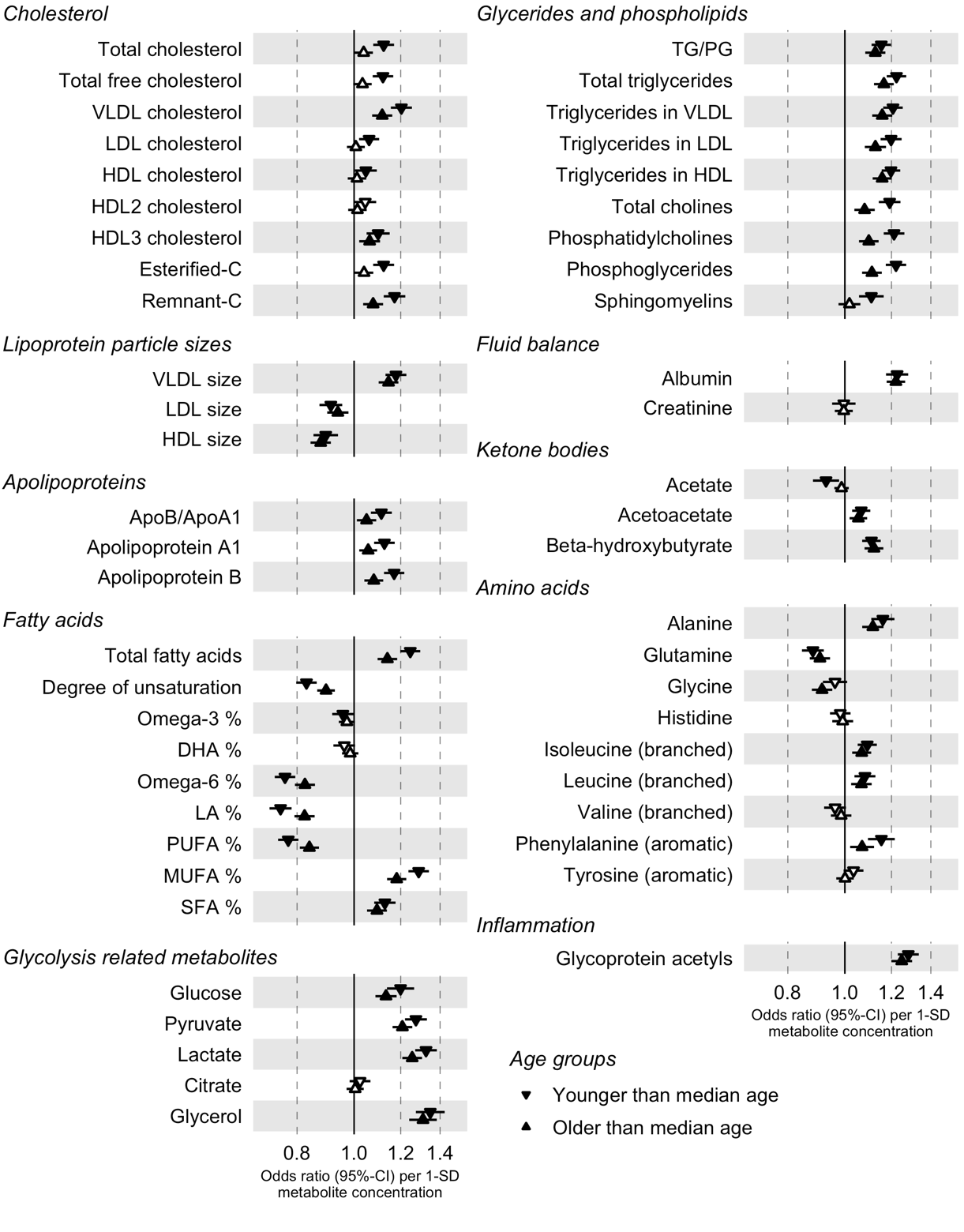


Filled triangle signifies FDR-corrected *P*<0.05. Associations are adjusted for age, sex, BMI, current smoking, diabetes, exercise, lipid medication, and cohort. MUFA, monounsaturated fatty acids; SFA, saturated fatty acids; TG, triglycerides; PG, phosphoglycerides; VLDL, very low density lipoprotein; LDL, low density lipoprotein; HDL, high density lipoprotein; Apo, apolipoprotein; C, cholesterol; DHA, docosahexaenoic acid; PUFA, polyunsaturated fatty acids; LA, linoleic acid.

**Figure S5.** Cross-sectional associations between lipoprotein measures related to 14 lipoprotein subclasses and hypertension.


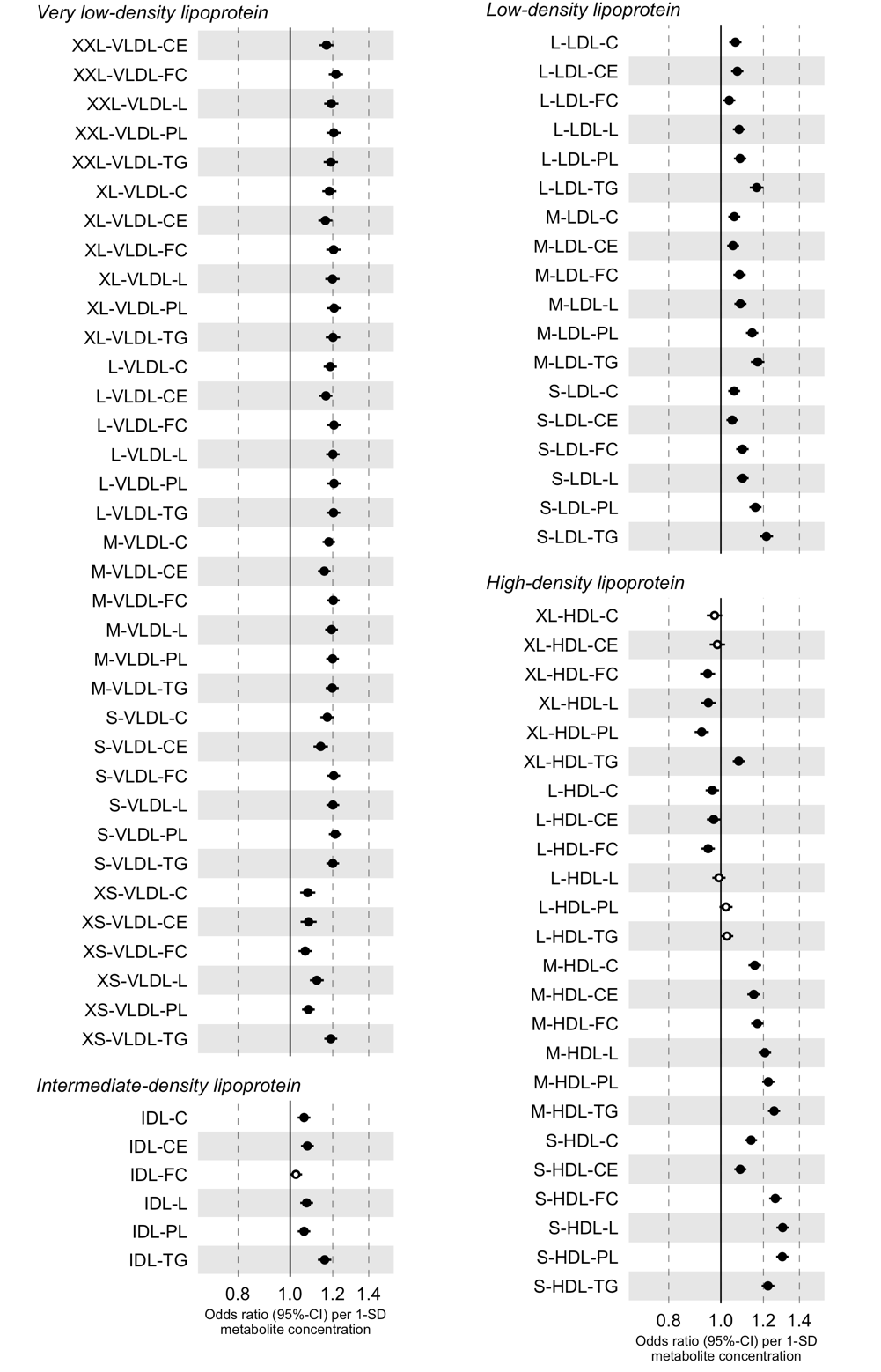


Filled circle signifies FDR-corrected P<0.05. Associations are adjusted for age, sex, BMI, current smoking, diabetes, antihypertensive medication, exercise, lipid medication, and cohort. C, Total cholesterol in lipoproteins; CE, Cholesterol esters in lipoproteins; FC, Free cholesterol in lipoproteins; HDL, high density lipoprotein; ILDL, intermediate-density lipoproteins; L, Total lipids in lipoproteins; LDL, low density lipoprotein; PL, Phospholipids in lipoproteins; TG, Triglycerides in lipoproteins. VLDL, very low density lipoprotein.

**Figure S6.** Longitudinal associations between baseline lipoprotein measures related to 14 lipoprotein subclasses and systolic blood pressure change (N=4197).


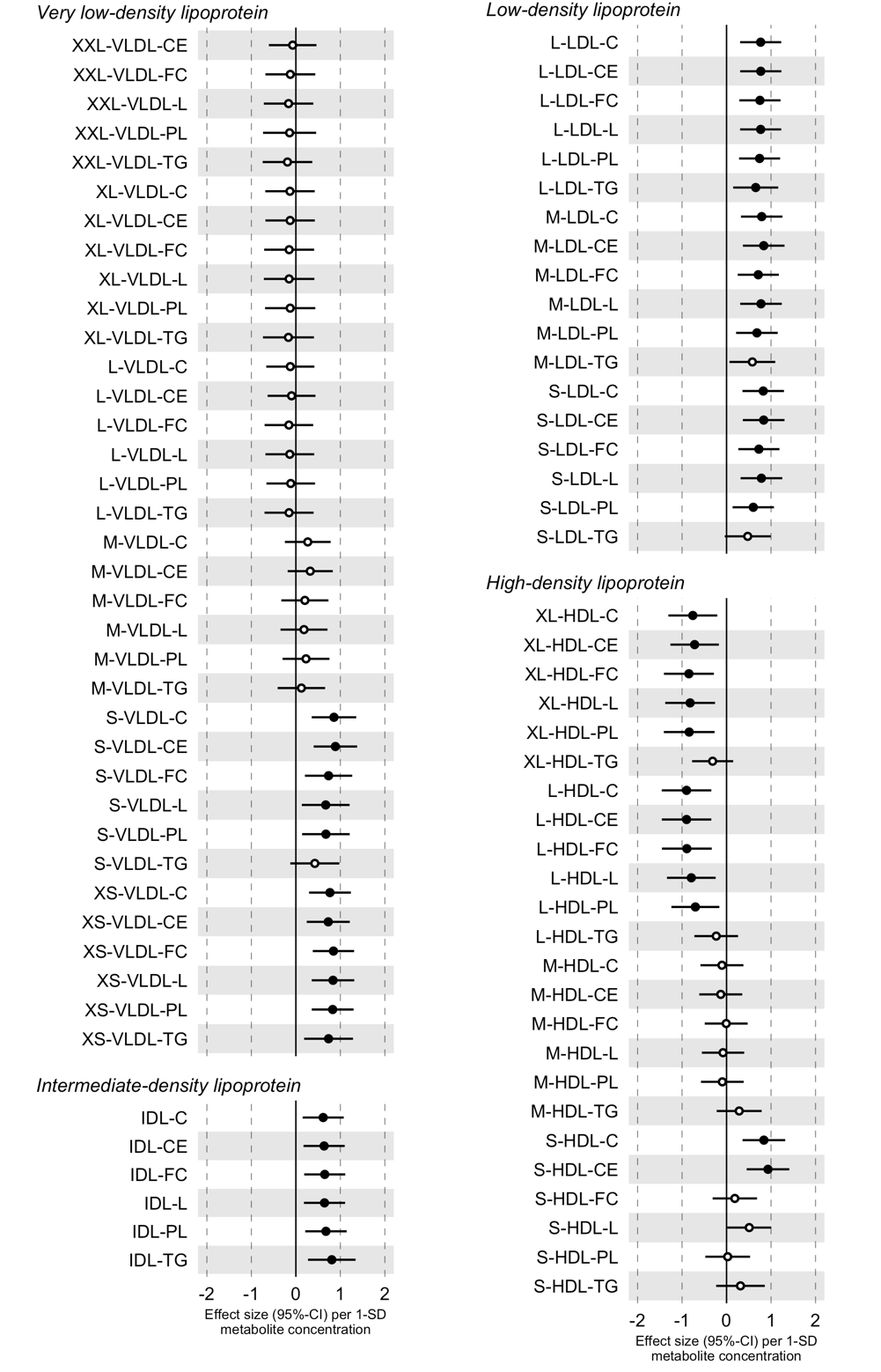


Filled circle signifies FDR-corrected *P*<0.05. Associations are adjusted for baseline systolic BP, age, sex, BMI, current smoking, diabetes, antihypertensive medication, exercise, lipid medication, and cohort. C, total cholesterol in lipoproteins; CE, cholesterol esters in lipoproteins; FC, free cholesterol in lipoproteins; HDL, high density lipoprotein; ILDL, intermediate-density lipoproteins; L, total lipids in lipoproteins; LDL, low density lipoprotein; PL, phospholipids in lipoproteins; TG, triglycerides in lipoproteins. VLDL, very low density lipoprotein.

**Figure S7.** Cross-sectional associations between metabolic measures and hypertension by cohort.


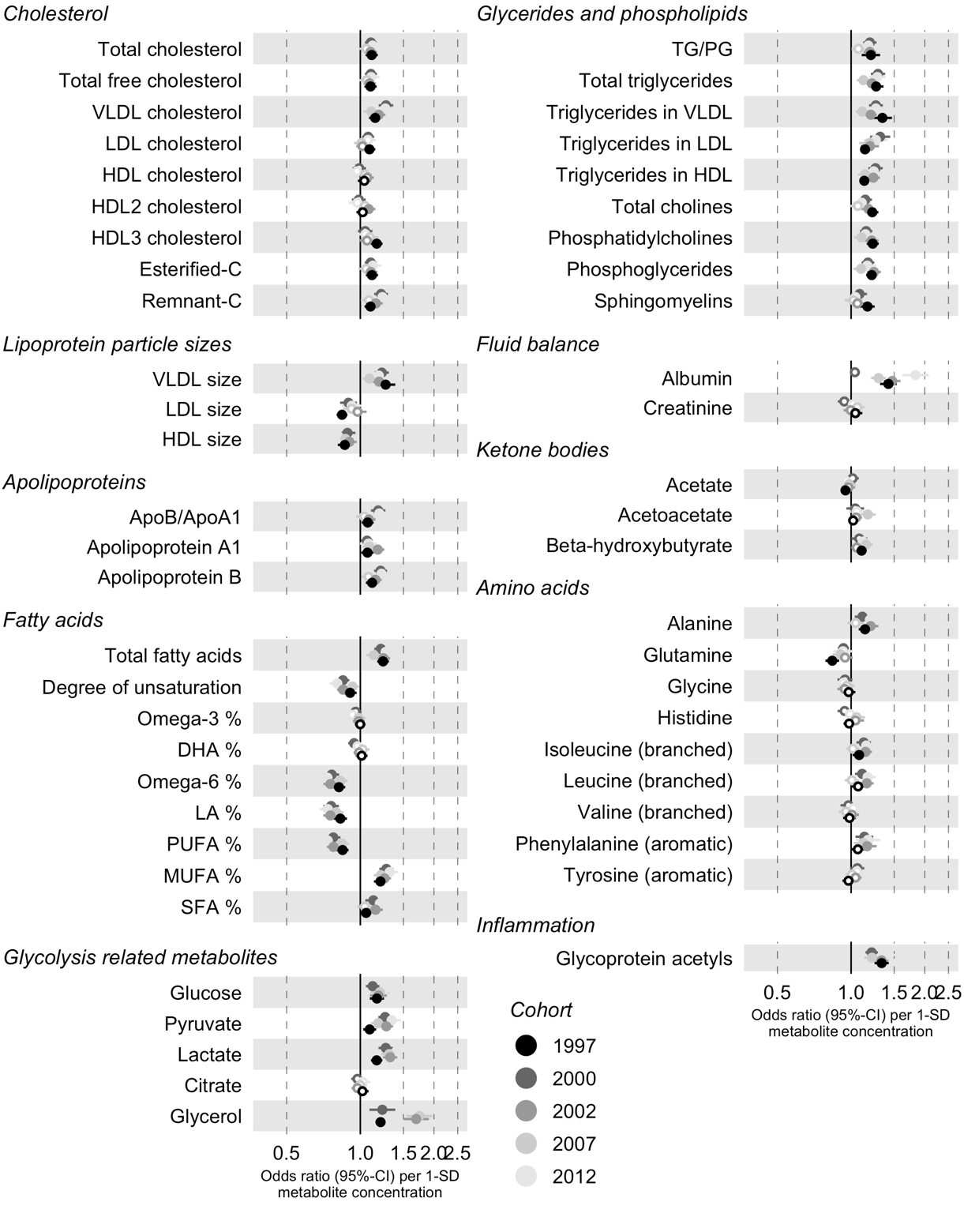


Filled circle signifies FDR-corrected *P*<0.05. Associations are adjusted for age, sex, BMI, current smoking, diabetes, exercise, and lipid medication. MUFA, monounsaturated fatty acids; SFA, saturated fatty acids; TG, triglycerides; PG, phosphoglycerides; VLDL, very low density lipoprotein; LDL, low density lipoprotein; HDL, high density lipoprotein; Apo, apolipoprotein; C, cholesterol; DHA, docosahexaenoic acid; PUFA, polyunsaturated fatty acids; LA, linoleic acid.

**Figure S8.** Meta-analysis for the cross-sectional associations between metabolic measures and continuous blood pressure variables.

**
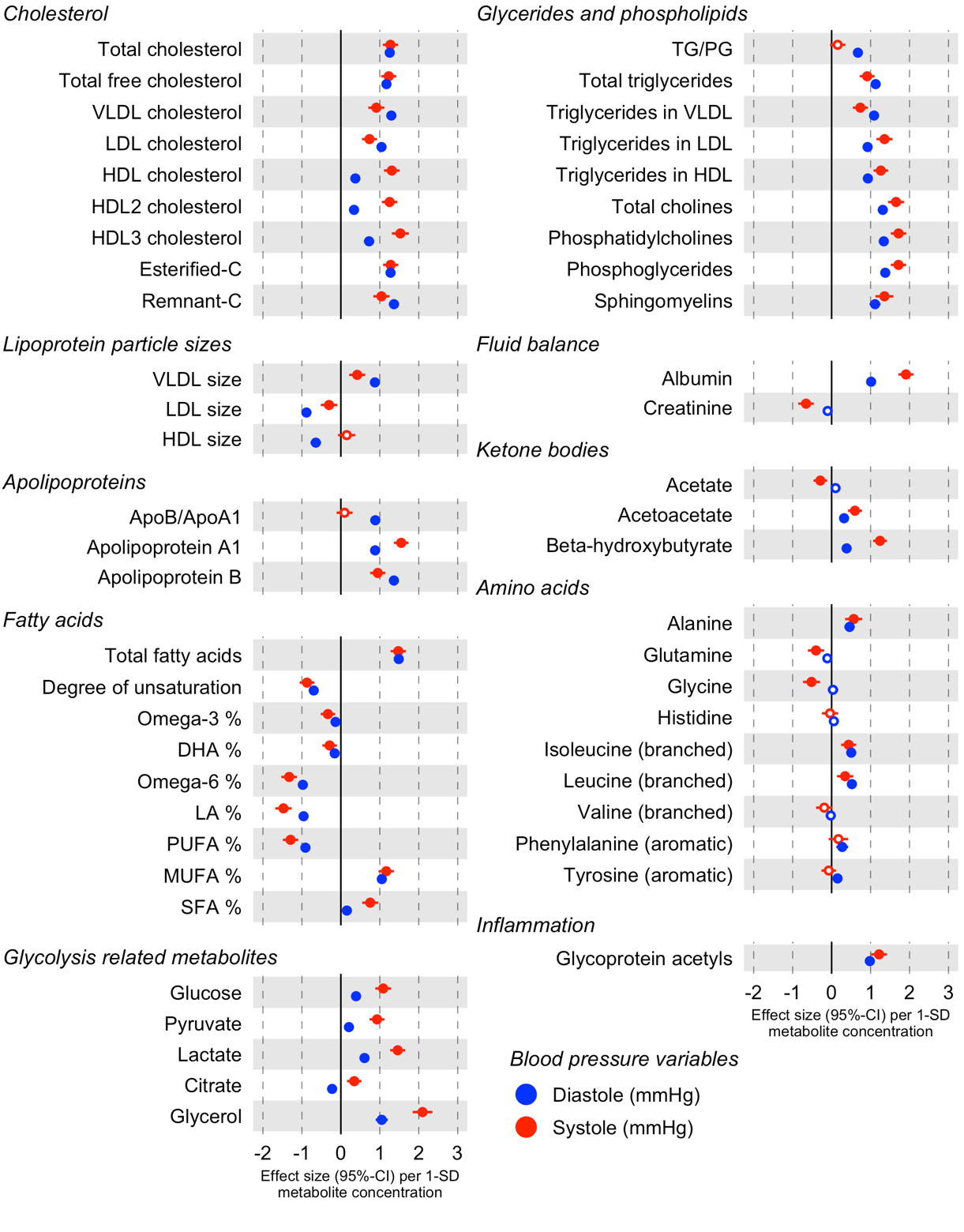
**

Filled circle signifies FDR-corrected *P*<0.05. Associations are adjusted for age, sex, BMI, current smoking, diabetes, antihypertensive medication, exercise, and lipid medication. MUFA, monounsaturated fatty acids; SFA, saturated fatty acids; TG, triglycerides; PG, phosphoglycerides; VLDL, very low density lipoprotein; LDL, low density lipoprotein; HDL, high density lipoprotein; Apo, apolipoprotein; C, cholesterol; DHA, docosahexaenoic acid; PUFA, polyunsaturated fatty acids; LA, linoleic acid.

**Figure S9.** Meta-analysis for the longitudinal associations between metabolic measures and continuous blood pressure variables.


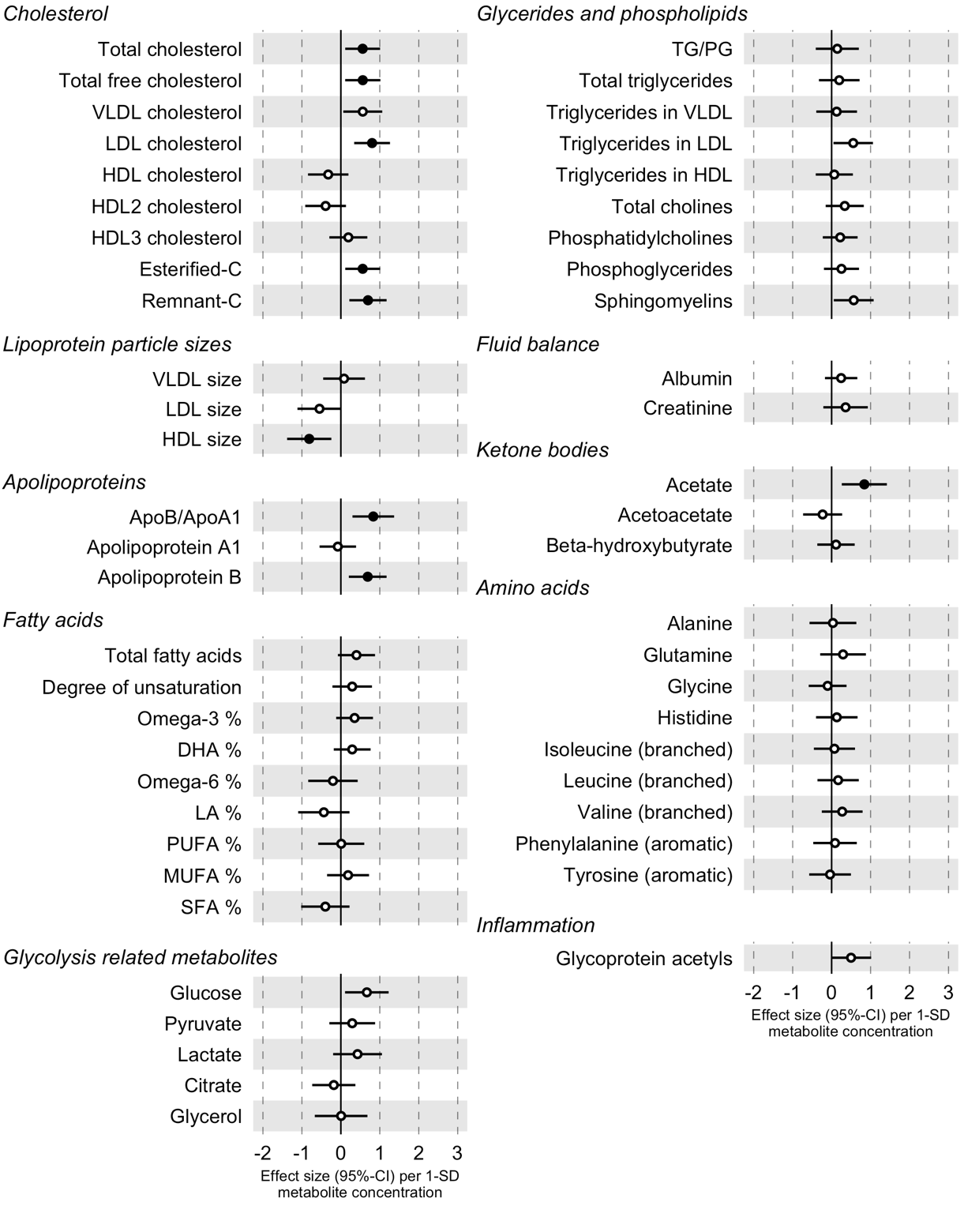


Filled circle signifies FDR-corrected *P*<0.05. Associations are adjusted for baseline systolic BP, age, sex, BMI, current smoking, diabetes, antihypertensive medication, exercise, lipid medication, and cohort. MUFA, monounsaturated fatty acids; SFA, saturated fatty acids; TG, triglycerides; PG, phosphoglycerides; VLDL, very low density lipoprotein; LDL, low density lipoprotein; HDL, high density lipoprotein; Apo, apolipoprotein; C, cholesterol; DHA, docosahexaenoic acid; PUFA, polyunsaturated fatty acids; LA, linoleic acid.
